# Supplementary material for: Prevalence of psychological distress, quality of life, and satisfaction among patients and family members following comprehensive genomic profiling testing: Protocol of the Quality of life for Cancer genomics and Advanced Therapeutics (Q-CAT) study
Source: PLoS One. 2023 May 26;18(5):e0283968. doi: 10.1371/journal.pone.0283968 (PMC10218744; doi:10.1371/journal.pone.0283968)
Supplement: S1 File — (DOCX) [file pone.0283968.s002.docx]

**Prevalence of psychological distress, quality of life, and satisfaction among patients and family members following comprehensive genomic profiling testing: Protocol of the Quality of Life for Cancer genomics and Advanced Therapeutics (Q-CAT) Study**

**Protocol**

Version draft - February 7, 2022

| Study Principal Investigator: | Takafumi Koyama, MD, National Cancer Center Hospital |
| --- | --- |
| Sponsors: |  |
| Protocol contributors: | Maiko Fujimori, PhD (National Cancer Center)  Makoto Hirata, MD, PhD (National Cancer Center Hospital)  Noriko Tanabe, PhD (National Cancer Center Hospital)  Toshio Shimizu, MD, PhD (National Cancer Center Hospital)  Noboru Yamamoto, MD, PhD (National Cancer Center Hospital)  Yosuke Uchitomi, MD, PhD (National Cancer Center Hospital) |
| Trial identifiers: | Japan Registry of Clinical Trials: RCT1030200039 |

**REVISION HISTORY**

| Version | Date | Amendment Text | Description |
| --- | --- | --- | --- |
| 1.1 | 2020/03/06 | Adjustment of description |  |
| 1.2 | 2020/03/10 | Adjustment of description |  |
| 1.3 | 2020/03/25 | Adjustment of description |  |
| 2.0 | 2020/07/01 | Consent withdrawal document addition  Co-Investigators addition  Adjustment of description |  |
| 2.1 | 2020/09/01 | Co-Investigators addition  Adjustment of description |  |
| 2.2 | 2021/01/27 | Extension of research period  Co-Investigators addition  Adjustment of description |  |
| 2.3 | 2021/04/20 | Extension of research period  Co-Investigators addition |  |
| 2.4 | 2021/07/21 | Co-Investigators addition  Adjustment of description |  |
| 2.5 | 2021/09/10 | Co-Investigators addition  Adjustment of description |  |
| 2.6 | 2022/02/07 | Adjustment of description |  |

**Table of Contents**

[1. TRIAL SUMMARY](#_Toc1)

[2. INTRODUCTION](#_Toc2)

[2.1. Background and rationale](#_Toc3)

[2.2. Objectives](#_Toc4)

[2.3. Trial design](#_Toc5)

[3. METHODS](#_Toc6)

[3.1. Study setting](#_Toc7)

[3.2. Eligibility criteria](#_Toc8)

[3.2.1. Inclusion criteria](#_Toc9)

[3.2.2. Exclusion criteria](#_Toc10)

[3.3. Interventions](#_Toc11)

[3.4. Outcomes](#_Toc16)

[3.5. Participant timeline](#_Toc17)

[3.6. Sample size](#_Toc18)

[3.7. Data collection](#_Toc24)

[3.7.1. Trial procedures and evaluations](#_Toc25)

[3.7.2. Retention](#_Toc26)

[3.8. Data management](#_Toc27)

[3.9. Statistical methods](#_Toc28)

[3.9.1. Outcomes](#_Toc29)

[3.9.2. Additional analyses](#_Toc30)

[3.9.3. Analysis population and missing data](#_Toc31)

[3.10. Data monitoring](#_Toc32)

[3.10.1. Formal committee](#_Toc33)

[3.10.2. Interim analysis](#_Toc34)

[3.11. Safety/harms](#_Toc35)

[4. ETHICS AND DISSEMINATION](#_Toc37)

[4.1. Research ethics approval](#_Toc38)

[4.2. Protocol amendments](#_Toc39)

[4.3. Informed consent process](#_Toc40)

[4.4. Confidentiality](#_Toc42)

[4.5. Declaration of interests](#_Toc43)

[4.6. Access to data](#_Toc44)

[4.7. Ancillary and post-trial care](#_Toc45)

[4.8. Dissemination policy](#_Toc46)

[4.8.1. Authorship](#_Toc47)

[5. STUDY ADMINISTRATION](#_Toc49)

[5.1. Key contacts](#_Toc50)

[5.2. Funders](#_Toc51)

[5.3. Roles and responsibilities](#_Toc52)

[5.3.1. Protocol contributors](#_Toc53)

[6. APPENDICES](#_Toc56)

[6.1. Informed consent materials](#_Toc57)

[6.2. Biological specimens](#_Toc58)

# 1. TRIAL SUMMARY

World Health Organization Registration Data Set

| Title | Prevalence of psychological distress, quality of life, and satisfaction among patients and family members following comprehensive genomic profiling testing: Protocol of the Quality of Life for Cancer genomics and Advanced Therapeutics (Q-CAT) Study |
| --- | --- |
| Primary registry and trial identifying number | Japan Registry of Clinical Trials: RCT1030200039 |
| Sources of monetary or material support | This work is supported by Japan Society for the Promotion of Science (JSPS) grant number 20K21742 and Daiwa Securities Health Foundation. |
| Central contact | Makoto Nishino, MD, PhD  National Cancer Center Hospital  5-1-1 Tsukiji. Chuo-ku, Tokyo, Japan  +81-3-3547-5201 Ext.3540 |
| Study officials/Investigators | Takafumi Koyama, MD  National Cancer Center Hospital  5-1-1 Tsukiji. Chuo-ku, Tokyo, Japan  +81-3-3547-5201, Ext.3036  takoyama@ncc.go.jp  Study Principal Investigator |
| Brief title | Protocol of the Quality of Life for Cancer genomics and Advanced Therapeutics (Q-CAT) Study |
| Acronym | Q-CAT |
| Countries of recruitment | Japan |
| Interventions | None |
| Key eligibility criteria | Age eligibility: No limit  Sex eligibility: Both  Accepts healthy volunteers: No  Inclusion criteria:  1. Patients at National Cancer Center Hospital  2. Patients pathologically diagnosed as malignant solid tumor  3. Patients planned to perform comprehensive cancer genome profiling  4. Patients with written informed consent  5. Family member of patients consented to this trial  6. Family member of patients who also attend the appointment for result of comprehensive cancer genome profiling  7. Family member of patients with written informed consent  Exclusion criteria:  1. Patients with difficulty writing and reading Japanese  2. Patients who was others judged by the investigator to be unsuitable for the study  3. Family member of patients with difficulty writing and reading Japanese  4. Family member of patients who was others judged by the investigator to be unsuitable for the study |
| Study design | Primary purpose: Supportive Care  Phase: Observational study |
| Date of first enrollment | May 18, 2020 |
| Target sample size | 500 |
| Recruitment status | Ongoing |
| Primary outcomes | Outcome: Prevalence of depression  Timeframe: at before comprehensive cancer genome profiling test, at the time of explanation of the result, at three times after the test, at the six months after the test |
| Secondary outcomes | Outcome: Anxiety, QOL, Symptom, Satisfaction, Quality of communication, Knowledge of comprehensive cancer genome profiling test, social support, Medical background, Social background, Medical information  Timeframe: at before comprehensive cancer genome profiling test, at the time of explanation of the result, at three times after the test, at the six months after the test |

# 2. INTRODUCTION

## 2.1. Background and rationale

On May 29th 2019, two cancer genomic profiling, CGP tests were reimbursed in Japan; which were "Onco GuideTM NCC Oncopanel system" and "Foundation One CDx". Recently the testing can be only allowed once in a lifetime for those who are advanced cancer patients with no standard therapy, or those who are advanced cancer patients finished standard therapies. The cost of the testing is very expensive that costs total ¥560,000 JPY, however the potential benefit patients enjoy from the cancer genomic profiling testing is individualized genomically appropriate therapeutics, on a word making precision oncology reality. In some malignancy, they already have companion diagnostic tools to survey one or two specific gene that allows to use genetically target therapy which has higher response rate and long survival benefit, such as EGFR-TKIs in non-small cell carcinoma (Maemondo et al., 2010, Mitsudomi et al., 2010). Even in these cancers with companion diagnostic tools, cancer genomic profiling testing sometimes allows to find rare but very effective actionable gene. Interestingly, about 10-20% of those who underwent testing CGP testing finally led to genetically matched therapies (Sunami et al., 2019). As these statistics shows that the ratio who receive benefit from CGP testing is not so much, the growing expectation and focus is not matching its clinical benefit ratio result in disappointment and mental burden both in cancer patients and their family members. There is research of CGP influence on mental health on breast hereditary cancer from Spain and on breast and cervical hereditary cancer from United States; we Japanese don't have any data on mental influence of CGP testing. Spanish research is multi-centered of 30cites with 187 candidates recruited with 4 times of follow up survey asking anxiety, mental burden (Esteban et al., 2018). Research from United States enrolled 232 patients and surveyed psychological impact, understanding (Lumish et al., 2017). Both of the research is focused on only hereditary cancers using germ cell cancer panels and suggest some negative impact toward mental health. They did not show the prevalence rate, clinical course, relative factors nor impact toward family members. Our research focus on not only germ cell mutation but also somatic mutation in CGPs. Moreover, 167 hospitals are selected to perform CGP in Japan, taking account of more limited hospital running clinical trials, there is surely difficulties to access the cites of clinical trials if patients luckily found to have rare actionable genomic alteration. According to Townsley & Selby（2005）'s report that reviewed the preventing factors of cancer patients who had difficulties to be enrolled to clinical trial, revealed that lack of social support had highly impacted against enrollment  It goes without saying that social support will be essential issue for patients with rare actionable mutation to be enrolled to genetically matched clinical trial. To make long story short, our study focusing to reveal the reality of patients and family members who underwent CGP testing toward longitudinal mental and psychological impact, social support, and QOL is essential for accurate and precise understanding of cancer patients with CGP testing.

## 2.2. Objectives

The aims of this study are to identify the prevalence of psychological distress, quality of life, and satisfaction with CGP testing among patients and their family members by gathering electronic patient-reported outcomes and qualitative data.

## 2.3. Trial design

This is an observational study. Participants are asked to undergo questionnaire, otherwise there is no intervention takes place.

# 3. METHODS

## 3.1. Study setting

This is a single institute observational study done in National Cancer Center Hospital   Japan.

## 3.2. Eligibility criteria

### 3.2.1. Inclusion criteria

1. Patients at National Cancer Center Hospital
2. Patients pathologically diagnosed as malignant solid tumor
3. Patients planned to perform comprehensive cancer genome profiling
4. Patients with written informed consent
5. Family member of patients consented to this trial
6. Family member of patients who also attend the appointment for result of comprehensive cancer genome profiling
7. Family member of patients with written informed consent

### 3.2.2. Exclusion criteria

1. Patients with difficulty writing and reading Japanese
2. Patients who were others judged by the investigator to be unsuitable for the study
3. Family member of patients with difficulty writing and reading Japanese
4. Family member of patients who was others judged by the investigator to be unsuitable for the study

## 3.3. Interventions

This is an observational study. Participants are asked to undergo questionnaire, otherwise there is no intervention takes place.

## 3.4. Outcomes

Primary outcome:

Depression

The nine items of Patient Health Questionnaire-9 (PHQ-9) [Spitzer et al., 1999] will be used to measure depression. Higher scores indicate greater possibility of major depression (a PHQ-9 score ≥10, the cut off score, had a sensitivity of 88% and a specificity of 88% for major depression).

Secondary outcomes:

Anxiety

The seven items of General Anxiety Disorder-7 (GAD-7) [Spitzer et al., 2006] will be used to measure anxiety. Higher scores indicate greater possibility of generalized anxiety disorder (a GAD-7 score ≥10 had a sensitivity of 89% and specificity of 82% for generalized anxiety disorder).

QOL

The 30 items of the European Organization for Research and Treatment of Cancer Quality of Life Questionnaire module Core 30 (EORTC QLQ−C30) [Aaronson et al., 1993] will be used to measure QOL. For global QOL scale and functional scales, higher score indicates a better level of function. For symptom-oriented scales, higher scores indicate more severe symptoms.

Symptoms

The nine items of the Edmonton Symptom Assessment System (ESAS) [Yokomichi et al., 2015] will be used to measure symptoms. High scores indicate greater morbidity.

Qualitative interviews

All participants with a PHQ-9 score above cut-off, and a small convenience-selected subset of participants will be invited at baseline and at each follow-up time-point to participate in structured interviews based on SCID-IV [JAMA, 2015].

Knowledge

A 10-item, multiple choice, study-developed questionnaire will be used to assess knowledge of the purpose of CGP testing, likely frequency of informative results, cancers in which informative results are more likely to be found, availability of tailored treatment options, and source of genetic knowledge. Scores will be summed from 0 to 10, with high scores indicating greater knowledge.

Satisfaction

The five items of the Patient Satisfaction Questionnaire (PSQ) will be used to measure satisfaction. Lower scores indicate higher satisfaction. [Blanchard et al., 1986]

Quality of communication

The 11 items on the quality of communication in the Health Information National Trends Survey (HINTS) will be used to measure quality of communication. High scores indicate greater communication.  [Smith et al., 2015]

Social support

The seven items of The Multidimensional Scale of Perceived Social Support (MSPSS) will be used to measure quality of communication. High scores indicate greater social support. [Zimet et al., 1988]

Demographic data

Age, gender, marital status, education level, occupation, and family history of cancer will be collected by patient report at baseline.

Disease details

Data available from the patient’s electronic health record at the National Cancer Center Hospital, including family history, primary site, detailed staging, Eastern Cooperative Oncology Group (ECOG) performance status, treatment and co-morbidities, will be collected at baseline.

## 3.5. Participant timeline


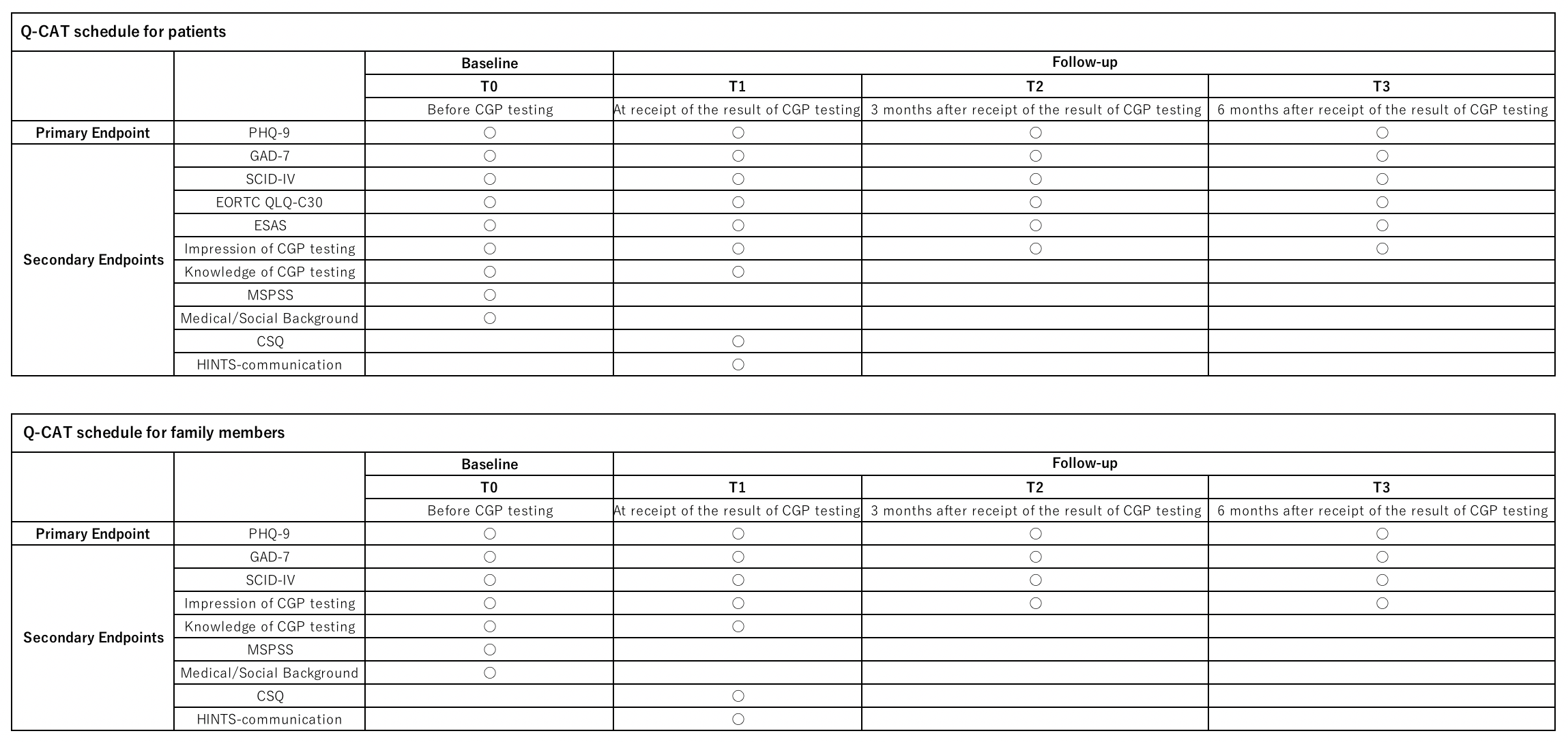


## 3.6. Sample size

The Q-CAT study plans to recruit 300 patients and 200 family members over a one-year period. These sample sizes were estimated based on the fact that an average of 33 patients were tested from September to October 2019, suggesting that 396 patients will be tested over a one-year period in our institute. Given an estimated participation rate in our QCAT trial of 75%, we determined a sample size of 300. For family members, an estimated sample size of 192 was chosen, based on an accompaniment rate by family members of patients visiting our outpatient clinic of 80%, with a consent rate of 80%.

## 3.7. Data collection

### 3.7.1. Trial procedures and evaluations

The Q-CAT (QOL for Cancer Genomics and Advanced Therapeutics among Patients and Their Family Members) study is being conducted as a psycho-social/ethical study with long span of four times; at the time of consent of CGP testing (T0), at receipt of the result of CGP testing (T1), three months after receipt of the result of CGP testing (T2), six months after receipt of the result of CGP testing (T3). By following candidate for enough duration of time, we could evaluate both immediate and gradual reality in psychosocial influence of CGP testing toward patients and their family members.

### 3.7.2. Retention

**Participant Withdrawal**

Participants, both patients and family members, may withdraw from the study for any reason at any time. The investigator also may withdraw participants from the study in order to protect their safety and/or if they are unwilling or unable to comply with required study procedures after consultation with protocol Chair.

## 3.8. Data management

Participants will be asked to complete a questionnaire using an electronic patient reported outcome (ePRO) application software or hard copy at baseline (T0), 1 to 4 weeks after receiving the results of CGP testing (T1; approximately 2–3 months post baseline), 3 months (T2) after T1, and 6 months (T3) after T1. These timeframes have been chosen to allow the impact of the test results to be captured in both the short term (when a treatment decision is being made) and longer term (while undergoing or close to completion of the targeted therapy, if adopted). All patients with a PHQ-9 score at the cutoff point of 10 or above (total score) as well as a small subset of participants (around 20–40) selected as a convenience sample who score below this cutoff point will be interviewed using the Structured Clinical Interview for DSM-V-RV (SCID), which includes items on mood disorders, neurotic, stress-related and somatoform disorders, and behavioral syndromes associated with physiological disturbances and physical factors, at each assessment time-point to determine the 4-week and lifetime prevalence rates of comorbid mental disorders

## 3.9. Statistical methods

### 3.9.1. Outcomes

To examine the possibility of selection bias, a comparative analysis of respondents and non-respondents will be conducted based on demographic and clinical data. Estimates of the prevalence of depression, anxiety, and other mental comorbidity in the target population will be reported as unadjusted raw rates observed in the total sample. Descriptive statistical analysis will be conducted for all data. Temporal changes in scales will be investigated by calculating differences between time-points. Comparisons of demographic and other factors will be conducted using the t-test and ANOVA, and Kruskal-Wallis test. Associations between variables will be examined using correlation analysis, chi-squared tests and regression analysis, depending on the presence or absence of depression and anxiety based on outcome scores. Multiple or logistic regression analysis will be used to adjust for the effects of confounders and to identify predictors of outcome. All analyses will be performed using IBM SPSS Statistics Version 26.

### 3.9.2. Additional analyses

N/A

### 3.9.3. Analysis population and missing data

N/A

## 3.10. Data monitoring

### 3.10.1. Formal committee

As this is a single institute observational study, we did not plan to set Data Monitoring Committee, DMC.

### 3.10.2. Interim analysis

N/A

## 3.11. Safety/harms

As our study is observational study with questionnaire, we don’t have therapeutical interventions, therefore we do not assume harms nor adverse event. However, during completing our questionnaire with ePRO, the participant will have potential to face difficulty of mental uncomfortableness. In such cases we would immediately consult our psycho-oncology teams.

# 4. ETHICS AND DISSEMINATION

## 4.1. Research ethics approval

This study has been approved by the National Cancer Center Institutional Review Board (2019-315). The results will be published in a scientific peer-reviewed journal, and findings will be disseminated at the domestic and international levels.

## 4.2. Protocol amendments

## 4.3. Informed consent process

Patients will provide consent for the study to Q-CAT staff at the same time they give consent to the CGP or within one week thereafter. Thus, at baseline they will have already considered and given consent for CGP testing but will not have received the results of CGP testing. Participants will be asked to complete a questionnaire using an electronic patient reported outcome (ePRO) application software or hard copy at baseline (T0), 1 to 4 weeks after receiving the results of CGP testing (T1; approximately 2–3 months post baseline), 3 months (T2) after T1, and 6 months (T3) after T1. These timeframes have been chosen to allow the impact of the test results to be captured in both the short term (when a treatment decision is being made) and longer term (while undergoing or close to completion of the targeted therapy, if adopted).

### 4.3.1. Ancillary studies

N/A

## 4.4. Confidentiality

Study participant will be anonymized with a research number. Research number and personal identification information are only connected by correspondence table, and the table is managed by personal information manager. Only anonymized data will be collected and be used in this study. All data are symbolized, digitized and stored primary in a secure external database and finally in the center. Personal information manager manage data in personal computer isolated from outside, take safety measures against information leaks by storing computers in a high-security room. In addition, the results of the research will not be reported or published in a manner that identifies individuals.

## 4.5. Declaration of interests

MN has reported honoraria from AstraZeneca, Bristol-Myers Squibb, Boehringer Ingelheim Japan, Chugai, Eli Lilly, MSD, Novartis, Pfizer, ONO, and Taiho, outside the submitted work.

TK has reported receiving personal fees from Chugai and Sysmex, and grants from PACT Pharma outside the submitted work.

TS has a consultancy/advisory role for Takeda Oncology, and has obtained research funding for his institution from Novartis, Eli Lilly, AbbVie, AstraZeneca, Eisai, Millennium-Takeda, Bristol-Myers Squibb, Incyte, Astellas Pharma, Symbio Pharmaceuticals, 3D-Medicine, Chordia Therapeutics, Five Prime, PharmaMar, and Daiichi-Sankyo outside the submitted work; and acts as a Scientific Committee Member for Phase 1 Trials in Hong Kong under the Consortium on Harmonization of Institutional Requirements for Clinical Research (CHAIR), Hong Kong, HKSAR China.

NY has a consultancy/advisory role with Eisai, Takeda Oncology, Otsuka, Boehringer Ingelheim, Cimic and Chugai, and has obtained research funding for his institution from Astellas Pharma, Chugai, Eisai, Taiho, Bristol-Myers Squibb, Pfizer, Novartis, Eli Lilly, AbbVie, Bayer, Boehringer Ingelheim, Daiichi-Sankyo, Kyowa-Hakko Kirin, Takeda, ONO, Janssen Pharma, MSD, Merck, GSK, and Sumitomo Dainippon, outside the submitted work.

The remaining authors declare no competing interests.

## 4.6. Access to data

Study participant will be anonymized with a research number. Research number and personal identification information are only connected by correspondence table, and the table is managed by personal information manager. Only anonymized data will be collected and be used in this study. All data are symbolized, digitized and stored primary in a secure external database and finally in the center. Personal information manager manage data in personal computer isolated from outside, take safety measures against information leaks by storing computers in a high-security room. In addition, the results of the research will not be reported or published in a manner that identifies individuals.

## 4.7. Ancillary and post-trial care

Information related to this research will be stored in accordance with the Standard Operating Procedures: SOP established by the institute. Information and research materials shall be preserved for 10 years from the publication of the study papers.

## 4.8. Dissemination policy

### 4.8.1. Authorship

MN, TK, MF, MH, NT, TS, NY, YU were responsible for the conception and initial study design, and for refining the study design. MN, TK, MF, NY and YU will be responsible for coordinating the acquisition of study data. MN, TK, MF will be responsible for statistical analysis, and interpretation and presentation of study data. MN, TK, MF, MH, NT, TS, NY, YU were involved in drafting the present manuscript and have read and approved the final manuscript.

# 5. STUDY ADMINISTRATION

## 5.1. Key contacts

**Study Principal Investigator**

Takafumi Koyama, MD

National Cancer Center Hospital

5-1-1 Tsukiji. Chuo-ku, Tokyo, Japan

+81-3-3547-5201, Ext.3036

takoyama@ncc.go.jp

**Central contact**

Makoto Nishino, MD, PhD

National Cancer Center Hospital

5-1-1 Tsukiji. Chuo-ku, Tokyo, Japan

+81-3-3547-5201, Ext.3540

## 5.2. Funders

This work is supported by Japan Society for the Promotion of Science (JSPS) grant number 20K21742 and Daiwa Securities Health Foundation.

## 5.3. Roles and responsibilities

### 5.3.1. Protocol contributors

MN, TK, MF, MH, NT, TS, NY, YU were responsible for the conception and initial study design, and for refining the study design. MN, TK, MF, NY and YU will be responsible for coordinating the acquisition of study data. MN, TK, MF will be responsible for statistical analysis, and interpretation and presentation of study data. MN, TK, MF, MH, NT, TS, NY, YU were involved in drafting the present manuscript and have read and approved the final manuscript.

# 6. APPENDICES

## 6.1. Informed consent materials

This study is observational study and possesses no interventions. We also don’t use any human biological specimens. However, as we handle special care-required personal information, we obtain consent using the written document from participants.

The consent document outlining this research will be used to explain this study, and appropriate written consent will be obtained from the patient and their family members. Participation in this research shall be of the individual’s own free will, and it shall be explained in writing that even after consenting to this research, the individual may withdraw at any time, and that there will be no disadvantages due to non-participation or withdrawal. Also, explain in writing that the results obtained will be processed statistically and that personal privacy will be strictly protected. In addition. Once participants are enrolled, they will not be de-registered (deleted from the database) unless there is a withdrawal of consent, including a refusal to use the data for research purposes.

## 6.2. Biological specimens

## N/A
