# Supplementary material for: Prevalence of psychological distress, quality of life, and satisfaction among patients and family members following comprehensive genomic profiling testing: Protocol of the Quality of life for Cancer genomics and Advanced Therapeutics (Q-CAT) study
Source: PLoS One. 2023 May 26;18(5):e0283968. doi: 10.1371/journal.pone.0283968 (PMC10218744; doi:10.1371/journal.pone.0283968)
Supplement: S2 File — (DOCX) [file pone.0283968.s003.docx]

がん遺伝子パネル検査における患者および同伴者の

QOLに関する研究

　　　　（研究責任者）国立がん研究センター中央病院　先端医療科　　　　小山　隆文

　　　（研究事務局）国立がん研究センター中央病院　先端医療科　　　　西野　誠

　　　　　　　　　　　　がん対策研究所 支持・サバイバーシップTR研究部　藤森　麻衣子

［2020年02月21日　Ver1.0 作成］

［2020年03月06日　Ver1.1 作成］

［2020年03月10日　Ver1.2 作成］

［2020年03月14日　Ver1.3 作成］

［2020年03月25日　Ver1.4 作成］

［2020年07月01日　Ver2.0 作成］

［2020年09月01日　Ver2.1 作成］

［2021年01月27日　Ver2.2 作成］

［2021年04月20日　Ver2.3 作成］

［2021年07月21日　Ver2.4 作成］

［2021年09月10日　Ver2.5 作成］

［2022年02月07日　Ver2.6 作成］

目　次

1. 概要
2. 研究の名称
3. 研究の実施体制
4. 研究の目的及び背景
5. 研究対象者の撰定方針
6. 期間・方法
7. インフォームド・コンセント
8. 個人情報等の取扱い
9. 研究参加のリスク・ベネフィット
10. 試料・情報の保管および廃棄の方法
11. 研究期間の長への報告内容及び方法
12. 研究に係る資金と利益相反に関する状況
13. 研究に関する情報公開の方法
14. 研究対象者等及びその関係者からの相談等への対応
15. 研究対象者等に経済的負担又は謝礼がある場合には、その旨及びその内容
16. 侵襲(軽微な侵襲を除く)を伴う研究の場合には、重篤な有害事象が発生した際の対応
17. 当該研究によって生じた健康被害に対する補償の有無及びその内容
18. 研究対象者に係る研究結果(偶発的所見を含む)の取扱い
19. 委託する業務内容及び委託先の監督方法
20. 本研究において取得した試料及び情報の将来的な二次利用
21. 参考文献リスト
22. 改訂履歴

別紙１　説明文書・同意書

別紙２　本研究の手順

別紙３　質問票

# 0．概要

名称：がん遺伝子パネル検査における患者および同伴者のQOLに関する研究

研究体制：

研究責任者　国立がん研究センター中央病院　 先端医療科　 小山　隆文

研究事務局　国立がん研究センター中央病院　 先端医療科　 西野　誠

国立がん研究センターがん対策研究所 支持・サバイバーシップTR研究部 藤森　麻衣子

共同研究者：

国立がん研究センターがん対策研究所 支持・サバイバーシップTR研究部

国立がん研究センター中央病院　 精神腫瘍科 内富　庸介

遺伝子診療部門　　　 平田　真

遺伝子診療部門　 田辺　記子

脳脊髄腫瘍科　　 高橋　雅道

頭頸部・食道内科 本間　義崇

腫瘍内科 須藤　一起

呼吸器内科 吉田　達哉

消化管内科 平野　秀和

肝胆膵内科 上野　秀樹

泌尿器科・後腹膜腫瘍科

前嶋　愛子

骨軟部腫瘍・リハビリテーション科

福島　俊

皮膚腫瘍科 緒方　大

小児腫瘍科 杉山　正仲

緩和医療科 石木　寛人

臨床検査科 角南　久仁子

先端医療科 清水　俊雄

看護部 中濱　洋子

看護部 佐々口　博子

看護部 小島　千恵美

看護部 稲村　直子

看護部 上原　智子

看護部 長橋　弘子

先端医療科 嶋岡　緑

国立がん研究センターがん対策研究所 支持・サバイバーシップTR研究部 佐藤　綾子

がん対策研究所 支持・サバイバーシップTR研究部 大歳　里沙

がん対策研究所 支持・サバイバーシップTR研究部 田中　郁美

個人情報管理者：

国立がん研究センター中央病院 先端医療科 山本　昇

目的：本研究の目的は、電子機器（タブレット端末）または紙面の質問票への回答による縦断調査により、がん遺伝子パネル検査を受ける前後のがん患者、同伴者の心理的苦痛およびQOLを明らかにし、患者背景、医師の検査の説明、検査結果等、関連要因を検討することである。本研究の結果は、がん遺伝子パネル検査に関連するがん対策を促進する基礎資料となることが期待される。

研究対象者の選定基準：

患者

（1）選択基準

1) 国立がん研究センター中央病院に通院している患者

2) がん診断の告知がなされている患者

3) がん遺伝子パネル検査を予定した患者

4）インフォームド・コンセントが得られる患者

（2）除外基準

　1) 日本語の読み書きが困難な患者

2) その他、研究責任者が不適当と判断した患者

同伴者

（1）選択基準

1) 本研究参加に同意した患者の同伴者

2) がん遺伝子パネル検査結果を聞く面談に同席する同伴者

3) インフォームド・コンセントが得られる同伴者

（2）除外基準

1) 日本語の読み書きが困難な同伴者

2) その他、研究責任者が不適当と判断した同伴者

研究の期間：研究許可日から2022年9月30日まで

研究のデザイン：前向きコホート研究

研究のアウトライン：

# 1．研究の名称

がん遺伝子パネル検査における患者および同伴者のQOLに関する研究

# 2．研究の実施体制

（研究責任者）

国立がん研究センター中央病院 先端医療科　 　 小山　隆文

（研究事務局）

国立がん研究センター中央病院　先端医療科　 　 　西野　誠

国立がん研究センターがん対策研究所 支持・サバイバーシップTR研究部 藤森　麻衣子

住所 〒104-0045 東京都中央区築地5-1-1

電話番号 03-3547-5201

（共同研究者）

国立がん研究センターがん対策研究所 支持・サバイバーシップTR研究部

国立がん研究センター中央病院　 精神腫瘍科 内富　庸介

　　　　　　　遺伝子診療部門　　　 平田　真

　　　　　　　遺伝子診療部門　　　 田辺　記子

　　　　　　　脳脊髄腫瘍科　 高橋　雅道

頭頸部・食道内科 本間　義崇

腫瘍内科 須藤　一起

呼吸器内科 吉田　達哉

消化管内科 平野　秀和

肝胆膵内科 上野　秀樹

泌尿器科・後腹膜腫瘍科

前嶋　愛子

骨軟部腫瘍・リハビリテーション科

福島　俊

皮膚腫瘍科 緒方　大

小児腫瘍科 杉山　正仲

緩和医療科 石木　寛人

臨床検査科 角南　久仁子

先端医療科 清水　俊雄

看護部 中濱　洋子

看護部 佐々口　博子

看護部 小島　千恵美

看護部 稲村　直子

看護部 上原　智子

看護部 長橋　弘子

先端医療科 嶋岡　緑

国立がん研究センターがん対策研究所 支持・サバイバーシップTR研究部 佐藤　綾子

がん対策研究所 支持・サバイバーシップTR研究部 大歳　里沙

がん対策研究所 支持・サバイバーシップTR研究部 田中　郁美

（個人情報管理者）

国立がん研究センター中央病院　先端医療科　 山本　昇

# 3．研究の目的及び背景

## 目的

本研究の目的は、電子機器（タブレット端末）または紙面の質問票への回答による縦断調査により、がん遺伝子パネル検査を受ける前後のがん患者、同伴者の心理的苦痛およびQOLを明らかにし、患者背景、医師の検査の説明、検査結果等、関連要因を検討することである。本研究の結果は、がん遺伝子パネル検査に関連するがん対策を促進する基礎資料となることが期待される。

## 背景

2019年5月29日、我が国でも次世代シークエンサーを用いた、がん遺伝子パネル検査が保険償還された。「OncoGuide^TM^ NCC オンコパネルシステム」と「FoundationOne CDx がんゲノムプロファイル」の2つが保険適用されたがん遺伝子パネル検査だが、いずれも施行は患者1人につき生涯1回で、標準的治療がない固形がん患者または局所進行や転移が認められ標準的治療が終了となった固形がん患者が対象となる。検査実施料は8000点、検査判断・説明料は4万8000点、合計の医療費は5万6000点（56万円）と高額なものであるが、プレシジョンメディシン（精密医療）、すなわち、患者の個人レベルで最適な治療方法を分析・選択し、治療を行うことを実現できる可能性がある。がん遺伝子変異を調べ治療に応用することは、肺癌、悪性黒色種などの一部のがん種においてコンパニオン診断薬（通常１つまたは2つの遺伝子を調べる）を用いて行われているが、すべてのがん種においては行われていない。非小細胞肺がんではEGFR変異陽性患者に対して、EGFR阻害薬（Gefetinibなど）を使用することで、標準治療の殺細胞性抗癌剤と比べ、高い奏効率と長い無増悪生存期間を示すことが証明されている(Maemondo et al., 2010, Mitsudomi et al., 2010)。このようなコンパニオン診断薬のあるがん種においても、コンパ二オン診断薬では検出できないような稀な活性化遺伝子変異ががん遺伝子検査を行うことで判明し、その変異を治療標的とした治療薬で奏効を示すことが報告されている。その一方で、Actionableな遺伝子変異が必ずしもみつかるわけではないこと、そして、検査結果から治療につながる可能性が全体の1−2割程度と言われている(Sunami et al., 2019)ことから、その恩恵に預かれる集団はまだ多いとは言えず、大きな注目・期待に見合わず、患者に落胆や心理的苦痛が生じうる可能性も懸念されている。

我が国においては遺伝子パネル検査の心理的影響に関する報告は見られないが、海外の先行研究では、スペインにおける乳がんの家族性腫瘍に関する遺伝子検査を受ける方を対象とした調査とアメリカにおける乳がん、子宮頸がんの家族性腫瘍に関する遺伝子パネル検査を受ける方を対象とした調査の結果が報告されている。スペインの調査では30施設、187名のゲノムパネル検査を受けたがん患者を対象に、検査前の遺伝カウセリング後にベースライン調査、結果開示後1週間、3か月、12か月後にフォローアップ調査を実施し、検査結果に関する意向、がんに対する不安、心理的苦痛、不確実感を評価した結果、変異が明らかな場合、患者の心理的苦痛、不確実感への影響は見出されなかったが、変異が中程度である場合には、検査結果開示1年後に患者の心理的苦痛が高いことが指摘されている（Esteban et al., 2018）。また、アメリカの調査では乳がん、子宮がんの遺伝子検査を受けた232名を対象に遺伝性パネル検査の心理的なインパクト、理解、遺伝情報の利用状況を調査し、乳がん、子宮がんの家族歴を有する未がんの方は心理的インパクトが大きいこと、関連要因として若年、教育歴の低さ、遺伝に関する知識の乏しさが示唆された（Lumish et al., 2017）。いずれも家族性腫瘍に関する遺伝子パネル検査を受けるものが対象であり、本研究対象である、がん遺伝子パネル検査を受けることが可能な標準治療終了後の患者を対象とした研究はこれまで報告されていない。また、その結果により一部の患者の心理面に負の影響を及ぼす可能性が言及されているが、その有症率や臨床経過、関連要因は明らかではない。さらに、家族等同伴者への心理的影響も、患者同様に想定されるが、これもまた、これまで検討されていない。

我が国のがん遺伝子パネル検査の実施は、全国11施設のがんゲノム医療中核拠点病院、全国34施設のがんゲノム拠点病院および122施設のがんゲノム医療連携病院に限定されており、検査の結果、治験や臨床試験等治療法が見つかったとしても実施施設はさらに限られていて、患者の医療機関へのアクセス上の制限があり、患者のソーシャルサポートの有無によって、治療選択に差が生じている懸念もある。Townsley & Selby（2005）によれば、高齢がん患者の臨床試験への参加を妨げる要因について文献をレビューした結果、併存疾患の制限、身体機能に加え、ソーシャルサポートの欠如や登録のための時間やリソースの必要性が指摘されている。

以上から、がん遺伝子パネル検査施行における、不安・懸念などの心情の揺らぎ、ソーシャルサポートの実態を調査することは、がん患者の現状把握と今後の新たな支援・指針を得るために重要と考える。そこで本研究の目的は、以下の2点である。

１．がん遺伝子パネル検査、およびその結果が患者、同伴者の心理的苦痛、およびQOLに及ぼす影響を検討する。

２．患者背景、医師の検査の説明、検査結果等、がん遺伝子パネル検査を受けた患者、同伴者の心理的苦痛、QOLに関連する要因を検討する。

# 4．研究対象者の選定方針

**4-1.　患者**

## （1）選択基準

　　1) 国立がん研究センター中央病院に通院している患者

2) がん診断の告知がなされている患者

3) がん遺伝子パネル検査を予定した患者

4）インフォームド・コンセントが得られる患者

（各選択基準の設定理由）

1) 国立がん研究センター中央病院の患者を対象とするため

2) がん患者を対象とするため

3) がん遺伝子パネル検査の心理的影響を把握するため

4）同意が得られたもののみ解析するため

## （2）除外基準

1) 日本語の読み書きが困難な同伴者

2) その他、研究責任者が不適当と判断した同伴者

（各選択基準の設定理由）

1) 説明同意文書が日本語のため

2) その他の状況で不適当である事を避けるため

**4-2.　同伴者**

## （1）選択基準

　　1) 本研究参加に同意した患者の同伴者

2) がん遺伝子パネル検査結果を聞く面談に同席する同伴者

3) インフォームド・コンセントが得られる同伴者

（各選択基準の設定理由）

1)、2) 本研究の対象の患者の同伴者へのがん遺伝子パネル検査の心理的影響を把握するため

3）同意が得られたもののみ解析するため

## （2）除外基準

1) 日本語の読み書きが困難な同伴者

2) その他、研究責任者が不適当と判断した同伴者

（各選択基準の設定理由）

1) 説明同意文書が日本語のため

2) その他の状況で不適当である事を避けるため

# 5．研究の期間及び方法

## （1）研究の期間

研究許可日から2022年9月30日まで

（2021年9月30日まで登録、その後、１年間で解析とする）

## （2）研究のデザイン

前向きコホート研究

## （3）研究のアウトライン

①パネル検査提出時：説明・同意取得、調査実施

②パネル検査結果説明時：調査実施

③②から3か月後：調査実施

④②から6か月後：調査実施

## （4）予定する研究対象者数

患者300名、同伴者192名

算定根拠：保険承認後の9月から10月までの2か月間に、国立がん研究センター中央病院においてがん遺伝子パネル検査を受けた患者は66名、月平均33名であることから、1年間に33×12名の患者が検査を受けると推定できる。本研究への参加率を75％と想定し300例とした。主要評価項目（0-27点）の有症率（軽症5点以上、中等度以上10点以上）と信頼区間を下記の表にまとめた。

同伴者は、患者の80％、参加率を80％と想定し、192例とした。

## （5）研究の対象とする医薬品・医療機器、治療法等の情報や使用方法など

次世代シークエンサー(NGS)はランダムに切断された数千万–数億のDNA断片の塩基配列を同時並行的に決定することができる。１回のアッセイにより100Gbp（G:ギガ＝十億）を越える配列を高い精度で決定でき、さらに、双方向性のペアエンド法を併用することによって、ヒトゲノムのような、ゲノムサイズが大きく、反復配列を多く含むゲノムにも詳細な解析が可能にした。染色体転座やコピー数異常などのゲノム構造異常の解析ができる。読み取り深度を深めること（ディープシーケンシング）によって、頻度の少ない変異を検出することも可能となる。（下記はNGS検査の概要）

がん遺伝子パネル検査はこのNGSを臨床応用したものとなる。腫瘍組織・非腫瘍組織などの試料から抽出されたDNAを用いて、最新のデータに基づいて決定された治療効果や毒性などに関わると考えられる遺伝子群の体細胞遺伝子変異（塩基置換・欠失・増幅・転座など）、遺伝子発現、遺伝子多型を解析・同定する（体細胞遺伝子プロファイリング）検査である。（下記は検査の概要）


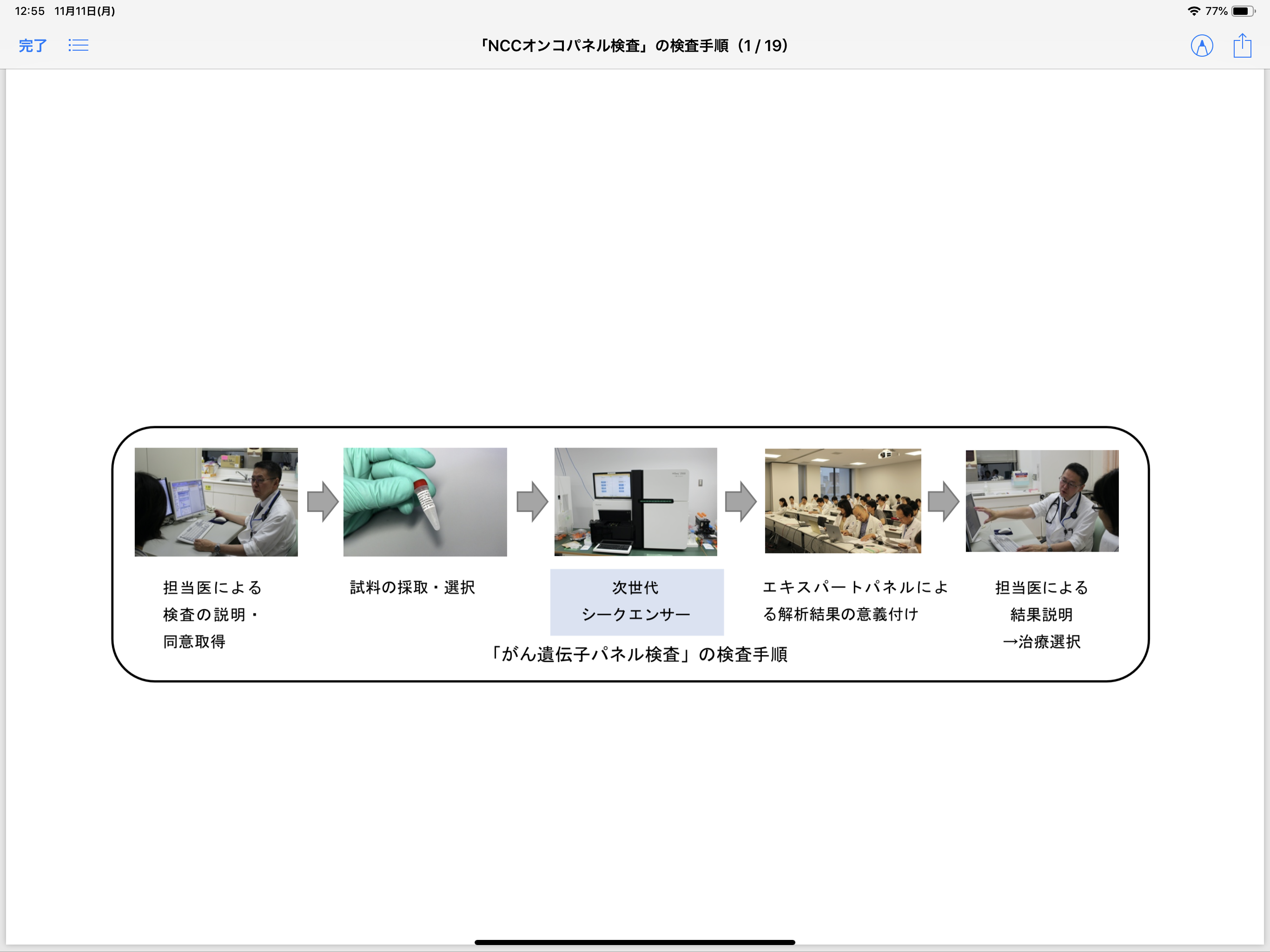


2019年5月29日に保険償還されたのは、「OncoGuide^TM^ NCC オンコパネルシステム」と「FoundationOne CDx がんゲノムプロファイル」の2つのがん遺伝子パネル検査だが、いずれも施行は患者1人につき生涯で1回のみで、標準治療がない固形がん患者または局所進行や転移が認められ標準治療が終了となった固形がん患者が対象となる。検査実施料は8000点、エキスパートパネルで検討された検査の判断・説明料は4万8000点、合計の医療費は5万6000点（56万円）と高額なものであるが、プレシジョンメディシン（精密医療）、すなわち、患者の個人レベルで最適な治療方法を分析・選択し、治療を行うことを実現できる可能性がある。遺伝子変異を調べ治療に応用することは、肺がん、悪性黒色種などの一部のがん種においてコンパニオン診断薬（通常1つまたは2つの遺伝子を調べる）を用いて行われているが、すべてのがん種においては行われていない。コンパニオン診断薬のあるがん種においても、コンパ二オン診断薬では検出できないような稀な活性化遺伝子変異ががん遺伝子検査を行うことで判明し、その変異を治療標的とした治療薬で奏効を示すことが報告されている。その一方で、Actionableな遺伝子変異が必ずしもみつかるわけではないこと、そして、検査結果から治療につながる可能性が全体の1−2割程度と言われていることから、その恩恵に預かれる集団はまだ多いとは言えない現状がある。

## （6）調査時間

電子機器（タブレット端末）または紙面の質問票への回答に要する時間は15-20分程度を見込んでいる。

面接調査の実施に要する時間は30分程度を見込んでいる。

## （7）評価項目及び評価方法

　　　（7）-1　評価項目

　　Primary endpoint：患者と同伴者の検査前、検査結果開示後、3か月後、半年後のうつ病の有症率

・Patient Health Questionnaire-9 (PHQ-9)：Spitzerら（1999）が開発したうつ病を評価するための9項目からなる自己記入式質問票である。評価は4件法（0：全くない-3：ほとんど毎日）が用いられ、総得点（0-27点）を算出し、重症度評価の基準は、0-4点：なし、5-9点：軽度、10-14点：中等度、15-19点：中等度-重度、20-27点：重度となっている。村松（2014）により日本語訳版が作成されている。中等度（10点）以上を示す患者と同伴者の割合をうつ病の有症率とする。また、副次的に、各重症度の割合、得点を記述する。

　　Secondary endpoint：患者・同伴者の以下の評価項目

　　・The Structured Clinical Interview for DSM-IV-TR　Axis I Disorders (SCID-IV)：Firstら（1997）が作成した精神科における患者の精神症状の評価および診断に用いるマニュアルである。日本語訳は高橋三郎ら（2003）によって行われている。DSM-IVの分類と診断墓準に習熟している臨床家や、訓練を受けた精神医療従事者が施行するものであるため、本研究においては、マニュアルに基づくトーレニングを行ったがん医療の経験を有する精神科医、臨床心理士（公認心理師）が施行する。本研究では、Mehnertら（2014）の報告に基づき、気分エピソード、気分障害（大うつ病性障害、双極性障害、小うつ病性障害、過去の大うつ病）、物質使用障害（アルコール、ニコチン依存、乱用）、不安障害（急性ストレス障害を含む）、適応障害の1か月有病率を評価する。PHQ-9にて10点以上（中等度以上）の患者と同伴者、および9点以下（軽度以下）の患者と同伴者の一部から診断面接を行う。

・General Anxiety Disorder-7 (GAD-7)：Spitzerら（2006）が開発した不安障害を評価するための7項目からなる自己記入式質問票である。評価は4件法（0：全くない-3：ほとんど毎日）が用いられ、総得点（0-21点）を算出する。村松（2014）により日本語訳版が作成されている。

・European Organization for Research and Treatment of Cancer Quality of Life Questionnaire module Core 30 (EORTC QLQ−C30)：欧州がん研究・治療機構によって開発されたがん患者の生活の質を評価する自己記入式質問票であり、症状や日常生活への支障の程度に関する30項目、28項目は4件法（1：まったくない-4：とても多い）、2項目は7件法（1：とても悪い-7：とてもよい）、機能の5尺度（身体、役割、認知、情緒、社会生活）、症状の3尺度(疲労感、疼痛、嘔気/嘔吐)と6単一項目（呼吸困難、不眠、食欲不振、便秘、下痢、経済的困難）、全般的QOLの1尺度で構成されている（Aaronson et al., 1993）。下妻ら（2001）により、日本語版が作成されている。

・Edmonton Symptom Assessment System Revised Japanese version（ESAS-r-J）：エドモントン症状評価システム（ESAS）は1991年にBrueraらが開発した、緩和医療の対象となる患者が頻繁に経験する9つの症状（痛み、だるさ、眠気、吐き気、食欲不振、息苦しさ、気分の落ち込み、不安、全体的な調子）をアセスメントするための自己記入式質問票である。ESAS-rは個々の症状を評価できるように10番目の症状の欄を空けている。今の症状に対する評価を11件法（0：なし-10：最もひどい）が用いて求めるものである。Yokomichiら（2018）により日本語版が作成されている。

・Client Satisfaction Questionnaire（CSQ）：医師が患者にどんなニードがあるかを知ろうとする、医師が患者との話し合いに積極的である、医師の患者との関わりあいの総合評価）、医師の説明、医師の患者への精神的なサポート）の５項目について、それぞれ0から10の11段階評価で質問票合計点を得る。

・検査結果開示時の医師のコミュニケーションの質：米国において実施されている健康情報に関する調査Health Information National Trends Survey (HINTS)（Smith et al., 2010）において評価されている医療者のコミュニケーションの質を評価する11項目を用いる。

・がん遺伝子パネル検査に関する知識：1)がん遺伝子パネル検査の目的、2)がん遺伝子パネル検査の利点、3)がん遺伝子パネル検査の限界、4)検査方法、5)がん遺伝子パネル検査結果の説明、6)検査のタイミング

・The Multidimensional Scale of Perceived Social Support (MSPSS)：Gresoryら（1990）が開発したソーシャルサポートを評価するための7項目からなる自己記入式質問票である。評価は7件法（0：まったくそう思わない（いない）-6：非常にそう思う）が用いられる。岩佐ら（2007）により日本語版が作成されている。

・医学的背景：確定診断日、がんの部位・組織型・病期、現在及び過去のがんの治療内容、Performance statusについてカルテから転記する。併存疾患について回答を求める。

・社会学的背景：年齢、性別、家族構成、教育年数、婚姻状況、雇用状況、世帯収入、ソーシャルサポート、自宅から病院までの通院の所要時間について本研究対象者に回答を求める。

・研究参加者の診療内容および医療利用の状況：研究参加者の診療の内容について診療録に記録された内容を収集する。がん遺伝パネル検査結果による遺伝子変異について、遺伝子解析実施/非実施、遺伝子変異の有/無/不明、治療法に結びつく遺伝子変異の有/無、その後の治療選択について遺伝子変異に伴い提示された治療法実施の有/無、その他治験等治療実施の有/無についてカルテから転記する。また、使用された薬剤、行われた医療行為、化学療法のレジメン、治療日、死亡日、死亡前1か月の入院日数、最終化学療法投与日から死亡までの日数などの情報を収集する。

他施設への調査が必要な場合には文書により情報提供を依頼する。
追跡調査を行うことについては、説明・同意文書に記載する。

　　　（7）-2　評価方法

がん遺伝子パネル検査の実施に対して同意をえた患者に対して調査者がリクルートを行う。その際、同伴者がいれば、同伴者もリクルートを行う。本試験への同意が得られた対象者に対して、質問票への回答を求める（検査前ベースライン調査）。検査前ベースライン調査の実施は、がん遺伝子パネル検査の同意から7日目までを許容するものとする。また、検査結果の開示後（１か月後までを許容）、3か月後（±1ヶ月を許容）、6か月後（±1ヶ月を許容）に同様の質問票への回答を求める。

調査スケジュールは以下の通りである。


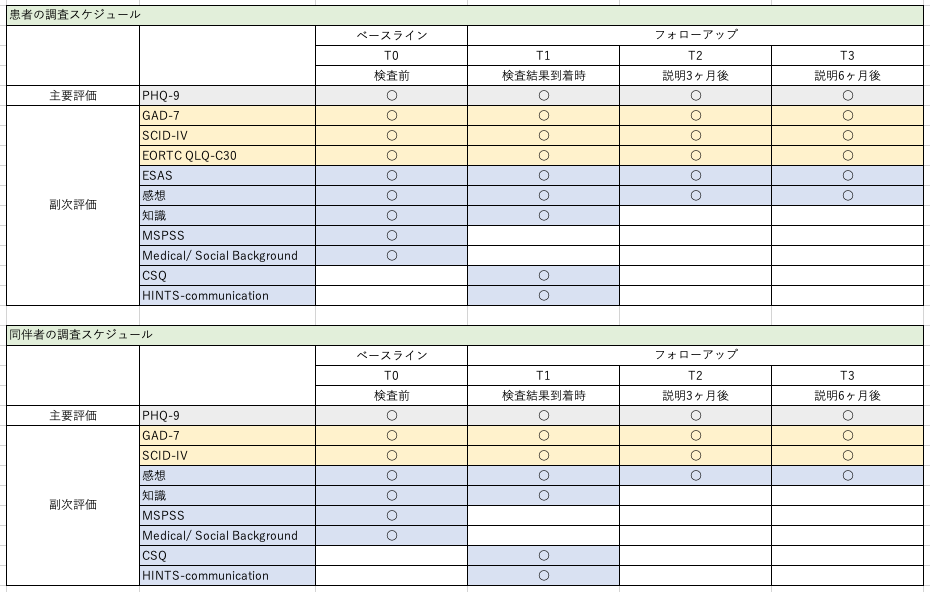


## （8）統計解析方法

主要評価、副次評価である自己記入式質問票、面接診断による評定値、医学的背景、社会的背景の記述統計量（平均値、標準偏差、中央値、最大値、最小値、95％信頼区間、閾値以上/以下の割合）を算出する。

各変数（2変数）間の関連を相関分析、χ二乗分析、t検定、ANOVA、Kruskal-Wallis検定、回帰分析により検討する。

有意水準は両側5％とする。

経時的に測定されているエンドポイントについては、各測定時点での結果を反応変数とし、欠測状況（欠測がある場合には可能であればその理由）を確認したうえで、全体の推移をグラフ化し各時点での要約統計量等を算出する。

## （9）研究実施に伴う遺伝カウンセリングの必要性と有無

がん遺伝子パネル検査の結果をもとにエキスパートにより必要と判断されれば、日常臨床で遺伝相談外来受診となるため、本研究に伴う必要性はない。遺伝子診療部門では「遺伝子パネル検査の二次的所見として生殖細胞系列変異が同定あるいは示唆された場合の対応方針」に基づきエキスパートパネルにおける判断ならびに遺伝相談外来対応を進める。

## （10）アンケート調査中止基準

以下の状況によってアンケート調査への協力が中止した場合をアンケート調査中止と定義する。研究者はアンケート調査中止に至った理由を研究責任者・研究事務局に報告する。研究者は患者および同伴者に対して、（11）同意撤回に記載する情報収集および利用の範囲に関して、原則確認をとる。

∙ 心理的負担等により、医師が患者へのアンケート調査中止を要すると判断した場合。ただし経過中に精神症状等が生じた場合にも、本研究と関連なく通常の臨床経過において生じたと判断される場合には、必ずしもアンケート調査中止とはしない。

∙ 研究期間中の患者の死亡。

∙ 登録後の患者状態の急速な増悪、プロトコール違反や不適格性の判明などがあった場合。

∙ 患者からアンケート調査中止の申し出が口頭または文書であった場合

## （11）同意撤回

本研究への同意撤回は、患者より主治医または研究者に対して、同意撤回の意志を文書または^＊^口頭で伝えることによってなされる。研究者は同意撤回を知りえた後に、本研究で規程している情報収集および利用に関する許容範囲 1)〜3）を患者および同伴者に確認する。その後、研究者は、研究責任者または研究事務局に同意撤回に関して連絡を行う。

1)追加アンケートへの同意撤回：追加のアンケート調査中止を意味する。それまでに収集したアンケート結果、臨床情報、以降のプロトコールに従った診療情報の収集に対して同意する。

2)追加情報収集に対する同意撤回：追加のアンケート調査中止し、同意撤回以降の診療情報収集も許容しない。同意撤回前のアンケート調査結果、収集した診療情報の利用を許容する。

3)本研究で収集した情報の利用に対する同意撤回：本研究に同意以降、収集されたすべての情報を許容しない

＊同意撤回に際して文書による意志表明が必要とすることは、かえって同意撤回に対する心理的障壁を高める（同意撤回しにくくなる可能性がある。被験者保護の観点から望ましくないと考えられることから、書式は予め作成されているが、同意撤回に際し、文書による意志表明は必須とせず、口頭によるものも有効とする。口頭による同意撤回に関しては、データベースに記録する。

# 6．インフォームド・コンセント

本研究は、介入を行わず人体から取得された試料を用いないが、要配慮個人情報を取得して研究を実施するため、「人を対象とする医学系研究に関する倫理指針」の第12の１(1)イ②（ⅰ）に従い、研究対象者から適切な同意を受ける。具体的には、研究の概要が記載された同意文書（別紙）を用いて患者および同伴者に説明を行い、適切な文書同意を取得する。

研究への参加は個人の自由意志によるものとし、本研究に同意した後でも随時撤回は可能であり、不参加・撤回による不利益は生じない事を文書にて説明する。又、得られた結果は統計学的に処理されるものであり、個人のプライバシーは厳重に守られる旨を文書を用いて説明する。また、データの研究利用の拒否を含む同意撤回があった場合を除いて、一度登録された患者は登録取り消し（データベースから抹消）はなされない。

# 7．個人情報等の取扱い（匿名化する場合にはその方法を含む。）

**（1）匿名化の方法及び安全管理措置**

研究対象者には研究用番号を付し匿名化を行う。研究用番号と個人識別情報（*氏名、カルテ番号*）を結ぶ対応表は、個人情報管理者の山本昇が管理し、匿名化した状態のデータのみを収集する。すべてのデータは記号化や数字化を行い、セキュリティーの高い外部データベースに一次的に保存し、最終的にはセンター（国立がん研究センター中央病院先端医療科科長室）で集約される。個人情報管理者は外部とは独立したパーソナルコンピュータでデータを管理し、個人情報管理者しか知らないパスワードを設定し、コンピュータをセキュリティーの厳重な部屋に保管することにより、情報の漏洩に対する安全対策を講じる。また、研究結果の報告、発表に関しては個人を特定される形では公表しない。

本研究におけるヒトゲノム・遺伝子解析の際の匿名化は、研究代表者が行い、その対応表は研究代表者が保管する。遺伝相談外来担当医が診療として行う「特定の遺伝性腫瘍に関する遺伝学的検査」結果とは異なり、本研究においては二次的所見であるため、研究代表者が遺伝学的診断を担当する遺伝相談外来受診者の対応表の一覧表を電子ファイルとして作成し、「国立研究開発法人国立がん研究センター中央病院診療録及び診療諸記録の電子保存に関する運用規程」を遵守し、インターネットに接続されていないPCおよびコミュニケーション系ファイルサーバー（その後継システム）等を用いて保管・運用する。

**（2）試料・情報の提供に関する記録の作成及び保存**

本研究は単施設研究であり、他機関への試料・情報の提供は行わない。

# 8．研究参加のリスク・ベネフィット

## （1）研究対象者に生じる負担と予想されるリスク、それらを最小化する方法

調査への回答には、20分程度の時間を要するため、時間的負担が生じるが、予め想定される調査回答所要時間、および途中で中断することが可能であることを提示し、同意が得られた者のみ研究対象とするとともに、研究対象者は調査を開始した後に中断することができる。一般的な質問調査であり、侵襲を伴わない介入のない調査研究であるため、対象に対する危険や不利益はないと考える。しかし、研究対象者から相談があった際には、研究計画を変更する、研究対象者を研究から脱落させる等、速やかに対応する。アンケート結果により、抑うつや不安障害などの医学的介入が必要な状態と判断された際には、当院の精神腫瘍科への紹介を考慮する。

## （2）予想される利益と負担・リスクを踏まえた総合評価

本研究への参加によって、研究対象者に直接の利益は生じない。研究成果により将来の医療の進歩に貢献できる可能性がある。

# 9．試料・情報（研究に用いられる情報に係る資料を含む。）の保管及び廃棄の方法

本研究に関する試料及び情報等は施設の定める手順書に従って保管する。論文等の形で発表された研究成果のもととなったアンケート結果等の研究資料は、当該論文等の発表から10年間の保存を原則とする。試料や標本などの有体物については5年間を原則とする。期限を過ぎた後も出来るだけ長期に保管することが推奨される。保管期間経過後、本研究に関する試料および情報を廃棄する場合は、匿名化したのち廃棄する。

# 10．研究機関の長への報告内容及び方法

本研究は、侵襲の無い観察研究であり研究期間が3年以内のため、研究責任者は研究実施状況報告を行わず、研究終了報告をもって研究期機関の長への報告とする。

ただし、研究を中止または終了した場合は、研究責任者は速やかに研究機関の長に報告を行う。また、以下に示す状況に該当するものが発生した場合も同様に、速やかに報告を行う。

・研究の倫理的妥当性若しくは科学的合理性を損なう事実若しくは情報又は損なうおそれのある情報であって研究の継続に影響を与えると考えられるものを得た場合

・研究の実施の適正性若しくは研究結果の信頼を損なう事実若しくは情報又は損なうおそれのある情報を得た場合

# 11．研究に係る資金と利益相反に関する状況

本研究の実施に際して、研究資金：令和2年度(2020年度) 挑戦的研究（萌芽）がん遺伝子パネル検査の心理社会的影響と関連する患者の期待と医師の態度に関する研究（研究代表者：内富庸介、課題管理番号： 20K21742）を使用する。研究者の利益相反は、国立がん研究センター利益相反委員会が管理する。

# 12．研究に関する情報公開の方法

本研究で得られた結果は、国内・国外の学会発表・専門学術誌で論文として公表する予定である。

# 13．研究対象者等及びその関係者からの相談等への対応

研究対象者等及びその関係者からの相談については、下記相談窓口にて対応する。相談は原則として電話で行うこととし、研究責任者が責任をもって対応する。

（相談窓口）

国立がん研究センター中央病院　先端医療科

住所： 〒104-0045　東京都中央区築地5-1-1

TEL： 03-3542-2511　（内線 3036）

研究事務局：西野　誠、藤森　麻衣子

研究責任者：小山　隆文

# 14．研究対象者等に経済的負担又は謝礼がある場合には、その旨及びその内容

本研究参加に伴い、研究対象者に経済的負担は発生しない。また、調査は個人差あるものの、１回30分程度の時間を要する。

# 15．侵襲（軽微な侵襲を除く。）を伴う研究の場合には、重篤な有害事象が発生した際の対応

本研究は侵襲（軽微な侵襲を除く。）を伴わない研究であり、重篤な有害事象の発生は想定されない。

# 16．当該研究によって生じた健康被害に対する補償の有無及びその内容

本研究は研究対象者に対する侵襲を伴わない研究であり、健康被害の発生は想定されない。

# 17．研究対象者に係る研究結果（偶発的所見を含む）の取扱い

本研究の実施に伴い、研究対象者等の健康や生命に関する重要な知見が得られる可能性はない。

# 18．委託する業務内容及び委託先の監督方法

本研究では外部組織に委託する業務はない。

# 19．本研究において取得した試料及び情報の将来的な二次利用

本研究で得られたデータを二次利用することがある。この場合は、個人を識別する情報と結びつかないよう匿名化した上、がん患者さんの生活の質の向上に役立てる目的に限り、データを利用する。具体的には、新たに研究計画書を作成し，国立がん研究センター研究倫理審査を受ける。なお、本研究において提供された個人情報の管理責任者は、研究責任者の小山隆文である。

# 20．参考文献リスト

1. Aaronson NK, Ahmedzai S, Bergman B, Bullinger M, Cull A, Duez NJ, et al. The European Organization for Research and Treatment of Cancer QLQ-C30: a quality-of-life instrument for use in international clinical trials in oncology. Journal of the National Cancer Institute. 1993; 85(5): 365-76.
2. Esteban I, Vilaró M, Adrover E, Angulo A, Carrasco E, Gadea N, Sánchez A, Ocaña T, Llort G, Jover R, Cubiella J, Servitja S, Herráiz M, Cid L, Martínez S, Oruezábal-Moreno MJ, Garau I, Khorrami S, Herreros-de-Tejada A, Morales R, Cano JM, Serrano R, López-Ceballos MH, González-Santiago S, Juan-Fita MJ, Alonso-Cerezo C, Casas A, Graña B, Teulé A, Alba E, Antón A, Guillén-Ponce C, Sánchez-Heras AB, Alés-Martínez JE, Brunet J, Balaguer F, Balmaña J. Psychological impact of multigene cancer panel testing in patients with a clinical suspicion of hereditary cancer across Spain. Psychooncology. 2018;27(6):1530-1537.
3. First M, Spitzer R, Gibbon M, Williams J. Structured Clinical Interview for DSM-IV-TR Axis I Disorder (SCID-IV). 精神科診断面接マニュアル使用の手引き・テスト用紙［第2版］高橋三郎監修、北村俊則、岡野禎治訳、日本評論社、2010年6月
4. 岩佐一、権藤恭之、増井幸恵、稲垣宏樹、河合千恵子、大塚理加、小川まどか、高山緑、蘭牟田洋美、鈴木隆雄．日本語版「ソーシャル・サポート尺度」の信頼性並びに妥当性―中高年者を対象とした検討―厚生の指標．2007；54（6）：26-33.
5. Lumish HS, Steinfeld H, Koval C, Russo D, Levinson E, Wynn J, Duong J, Chung WK. Impact of Panel Gene Testing for Hereditary Breast and Ovarian Cancer on Patients. J Genet Couns. 2017;26(5):1116-1129.
6. [Maemondo M](https://www.ncbi.nlm.nih.gov/pubmed/?term=Maemondo%20M%5BAuthor%5D&cauthor=true&cauthor_uid=20573926), [Inoue A](https://www.ncbi.nlm.nih.gov/pubmed/?term=Inoue%20A%5BAuthor%5D&cauthor=true&cauthor_uid=20573926), Kobayashi K, [Sugawara S](https://www.ncbi.nlm.nih.gov/pubmed/?term=Sugawara%20S%5BAuthor%5D&cauthor=true&cauthor_uid=20573926), Oizumi S, [Isobe H](https://www.ncbi.nlm.nih.gov/pubmed/?term=Isobe%20H%5BAuthor%5D&cauthor=true&cauthor_uid=20573926), [Gemma A](https://www.ncbi.nlm.nih.gov/pubmed/?term=Gemma%20A%5BAuthor%5D&cauthor=true&cauthor_uid=20573926), [Harada M](https://www.ncbi.nlm.nih.gov/pubmed/?term=Harada%20M%5BAuthor%5D&cauthor=true&cauthor_uid=20573926), [Yoshizawa H](https://www.ncbi.nlm.nih.gov/pubmed/?term=Yoshizawa%20H%5BAuthor%5D&cauthor=true&cauthor_uid=20573926), [Kinoshita I](https://www.ncbi.nlm.nih.gov/pubmed/?term=Kinoshita%20I%5BAuthor%5D&cauthor=true&cauthor_uid=20573926), [Fujita Y](https://www.ncbi.nlm.nih.gov/pubmed/?term=Fujita%20Y%5BAuthor%5D&cauthor=true&cauthor_uid=20573926), [Okinaga S](https://www.ncbi.nlm.nih.gov/pubmed/?term=Okinaga%20S%5BAuthor%5D&cauthor=true&cauthor_uid=20573926), [Hirano H](https://www.ncbi.nlm.nih.gov/pubmed/?term=Hirano%20H%5BAuthor%5D&cauthor=true&cauthor_uid=20573926), [Yoshimori K](https://www.ncbi.nlm.nih.gov/pubmed/?term=Yoshimori%20K%5BAuthor%5D&cauthor=true&cauthor_uid=20573926), [Harada T](https://www.ncbi.nlm.nih.gov/pubmed/?term=Harada%20T%5BAuthor%5D&cauthor=true&cauthor_uid=20573926), [Ogura T](https://www.ncbi.nlm.nih.gov/pubmed/?term=Ogura%20T%5BAuthor%5D&cauthor=true&cauthor_uid=20573926), [Ando M](https://www.ncbi.nlm.nih.gov/pubmed/?term=Ando%20M%5BAuthor%5D&cauthor=true&cauthor_uid=20573926), [Miyazawa H](https://www.ncbi.nlm.nih.gov/pubmed/?term=Miyazawa%20H%5BAuthor%5D&cauthor=true&cauthor_uid=20573926), [Tanaka T](https://www.ncbi.nlm.nih.gov/pubmed/?term=Tanaka%20T%5BAuthor%5D&cauthor=true&cauthor_uid=20573926), [Saijo Y](https://www.ncbi.nlm.nih.gov/pubmed/?term=Saijo%20Y%5BAuthor%5D&cauthor=true&cauthor_uid=20573926), [Hagiwara K](https://www.ncbi.nlm.nih.gov/pubmed/?term=Hagiwara%20K%5BAuthor%5D&cauthor=true&cauthor_uid=20573926), [Morita S](https://www.ncbi.nlm.nih.gov/pubmed/?term=Morita%20S%5BAuthor%5D&cauthor=true&cauthor_uid=20573926), [Nukiwa T](https://www.ncbi.nlm.nih.gov/pubmed/?term=Nukiwa%20T%5BAuthor%5D&cauthor=true&cauthor_uid=20573926). [North-East Japan Study Group](https://www.ncbi.nlm.nih.gov/pubmed/?term=North-East%20Japan%20Study%20Group%5BCorporate%20Author%5D). Gefitinib or chemotherapy for non-small-cell lung cancer with mutated EGFR. N Engl J Med 2010;362(25)2380-8.
7. Mehnert A, Brähler E, Faller H, Härter M, Keller M, Schulz H, Wegscheider K, Weis J, Boehncke A, Hund B, Reuter K, Richard M, Sehner S, Sommerfeldt S, Szalai C, Wittchen HU, Koch U. Four-week prevalence of mental disorders in patients with cancer across major tumor entities. J Clin Oncol. 2014;32(31):3540-6.
8. [Mitsudomi T](https://www.ncbi.nlm.nih.gov/pubmed/?term=Mitsudomi%20T%5BAuthor%5D&cauthor=true&cauthor_uid=20022809), [Morita S](https://www.ncbi.nlm.nih.gov/pubmed/?term=Morita%20S%5BAuthor%5D&cauthor=true&cauthor_uid=20022809), [Yatabe Y](https://www.ncbi.nlm.nih.gov/pubmed/?term=Yatabe%20Y%5BAuthor%5D&cauthor=true&cauthor_uid=20022809), [Negoro S](https://www.ncbi.nlm.nih.gov/pubmed/?term=Negoro%20S%5BAuthor%5D&cauthor=true&cauthor_uid=20022809), Okamoto I, [Tsurutani J](https://www.ncbi.nlm.nih.gov/pubmed/?term=Tsurutani%20J%5BAuthor%5D&cauthor=true&cauthor_uid=20022809), [Seto T](https://www.ncbi.nlm.nih.gov/pubmed/?term=Seto%20T%5BAuthor%5D&cauthor=true&cauthor_uid=20022809), [Satouchi M](https://www.ncbi.nlm.nih.gov/pubmed/?term=Satouchi%20M%5BAuthor%5D&cauthor=true&cauthor_uid=20022809), [Tada H](https://www.ncbi.nlm.nih.gov/pubmed/?term=Tada%20H%5BAuthor%5D&cauthor=true&cauthor_uid=20022809), [Hirashima T](https://www.ncbi.nlm.nih.gov/pubmed/?term=Hirashima%20T%5BAuthor%5D&cauthor=true&cauthor_uid=20022809), [Asami K](https://www.ncbi.nlm.nih.gov/pubmed/?term=Asami%20K%5BAuthor%5D&cauthor=true&cauthor_uid=20022809), [Katakami N](https://www.ncbi.nlm.nih.gov/pubmed/?term=Katakami%20N%5BAuthor%5D&cauthor=true&cauthor_uid=20022809), [Takada M](https://www.ncbi.nlm.nih.gov/pubmed/?term=Takada%20M%5BAuthor%5D&cauthor=true&cauthor_uid=20022809), [Yoshioka H](https://www.ncbi.nlm.nih.gov/pubmed/?term=Yoshioka%20H%5BAuthor%5D&cauthor=true&cauthor_uid=20022809), [Shibata K](https://www.ncbi.nlm.nih.gov/pubmed/?term=Shibata%20K%5BAuthor%5D&cauthor=true&cauthor_uid=20022809), [Kudoh S](https://www.ncbi.nlm.nih.gov/pubmed/?term=Kudoh%20S%5BAuthor%5D&cauthor=true&cauthor_uid=20022809), [Shimizu E](https://www.ncbi.nlm.nih.gov/pubmed/?term=Shimizu%20E%5BAuthor%5D&cauthor=true&cauthor_uid=20022809), [Saito H](https://www.ncbi.nlm.nih.gov/pubmed/?term=Saito%20H%5BAuthor%5D&cauthor=true&cauthor_uid=20022809), [Toyooka S](https://www.ncbi.nlm.nih.gov/pubmed/?term=Toyooka%20S%5BAuthor%5D&cauthor=true&cauthor_uid=20022809), [Nakagawa K](https://www.ncbi.nlm.nih.gov/pubmed/?term=Nakagawa%20K%5BAuthor%5D&cauthor=true&cauthor_uid=20022809), [Fukuoka M](https://www.ncbi.nlm.nih.gov/pubmed/?term=Fukuoka%20M%5BAuthor%5D&cauthor=true&cauthor_uid=20022809). West Japan Oncology Group. Gefitinib versus cisplatin plus docetaxel in patients with non-small-cell lung cancer harbouring mutations of the epidermal growth factor receptor (WJTOG3405): an open label, randomised phase 3 trial. Lancet Oncol 2010;11(2):121-8
9. 下妻晃二郎、江口成美：がん患者用QOL尺度の開発と臨床応用（I）．日医総研ワーキングペーパー　No. 56　2001年11月
10. Smith SG, Wolf MS, von Wagner C. Socioeconomic status, statistical confidence, and patient-provider communication: an analysis of the Health Information National Trends Survey (HINTS 2007). J Health Commun. 2010;15 Suppl 3:169-85.
11. [Sunami K](https://www.ncbi.nlm.nih.gov/pubmed/?term=Sunami%20K%5BAuthor%5D&cauthor=true&cauthor_uid=30742731), [Ichikawa H](https://www.ncbi.nlm.nih.gov/pubmed/?term=Ichikawa%20H%5BAuthor%5D&cauthor=true&cauthor_uid=30742731), [Kubo T](https://www.ncbi.nlm.nih.gov/pubmed/?term=Kubo%20T%5BAuthor%5D&cauthor=true&cauthor_uid=30742731), [Kato M](https://www.ncbi.nlm.nih.gov/pubmed/?term=Kato%20M%5BAuthor%5D&cauthor=true&cauthor_uid=30742731), [Fujiwara Y](https://www.ncbi.nlm.nih.gov/pubmed/?term=Fujiwara%20Y%5BAuthor%5D&cauthor=true&cauthor_uid=30742731), [Shimomura A](https://www.ncbi.nlm.nih.gov/pubmed/?term=Shimomura%20A%5BAuthor%5D&cauthor=true&cauthor_uid=30742731), [Koyama T](https://www.ncbi.nlm.nih.gov/pubmed/?term=Koyama%20T%5BAuthor%5D&cauthor=true&cauthor_uid=30742731), [Kakishima H](https://www.ncbi.nlm.nih.gov/pubmed/?term=Kakishima%20H%5BAuthor%5D&cauthor=true&cauthor_uid=30742731), [Kitami M](https://www.ncbi.nlm.nih.gov/pubmed/?term=Kitami%20M%5BAuthor%5D&cauthor=true&cauthor_uid=30742731), [Matsushita H](https://www.ncbi.nlm.nih.gov/pubmed/?term=Matsushita%20H%5BAuthor%5D&cauthor=true&cauthor_uid=30742731), [Furukawa E](https://www.ncbi.nlm.nih.gov/pubmed/?term=Furukawa%20E%5BAuthor%5D&cauthor=true&cauthor_uid=30742731), [Narushima D](https://www.ncbi.nlm.nih.gov/pubmed/?term=Narushima%20D%5BAuthor%5D&cauthor=true&cauthor_uid=30742731), [Nagai M](https://www.ncbi.nlm.nih.gov/pubmed/?term=Nagai%20M%5BAuthor%5D&cauthor=true&cauthor_uid=30742731), [Taniguchi H](https://www.ncbi.nlm.nih.gov/pubmed/?term=Taniguchi%20H%5BAuthor%5D&cauthor=true&cauthor_uid=30742731), [Motoi N](https://www.ncbi.nlm.nih.gov/pubmed/?term=Motoi%20N%5BAuthor%5D&cauthor=true&cauthor_uid=30742731), [Sekine S](https://www.ncbi.nlm.nih.gov/pubmed/?term=Sekine%20S%5BAuthor%5D&cauthor=true&cauthor_uid=30742731), [Maeshima A](https://www.ncbi.nlm.nih.gov/pubmed/?term=Maeshima%20A%5BAuthor%5D&cauthor=true&cauthor_uid=30742731), [Mori T](https://www.ncbi.nlm.nih.gov/pubmed/?term=Mori%20T%5BAuthor%5D&cauthor=true&cauthor_uid=30742731)^1^, [Watanabe R](https://www.ncbi.nlm.nih.gov/pubmed/?term=Watanabe%20R%5BAuthor%5D&cauthor=true&cauthor_uid=30742731), [Yoshida M](https://www.ncbi.nlm.nih.gov/pubmed/?term=Yoshida%20M%5BAuthor%5D&cauthor=true&cauthor_uid=30742731), [Yoshida A](https://www.ncbi.nlm.nih.gov/pubmed/?term=Yoshida%20A%5BAuthor%5D&cauthor=true&cauthor_uid=30742731), [Yoshida H](https://www.ncbi.nlm.nih.gov/pubmed/?term=Yoshida%20H%5BAuthor%5D&cauthor=true&cauthor_uid=30742731), [Satomi K](https://www.ncbi.nlm.nih.gov/pubmed/?term=Satomi%20K%5BAuthor%5D&cauthor=true&cauthor_uid=30742731), [Sukeda A](https://www.ncbi.nlm.nih.gov/pubmed/?term=Sukeda%20A%5BAuthor%5D&cauthor=true&cauthor_uid=30742731), [Hashimoto T](https://www.ncbi.nlm.nih.gov/pubmed/?term=Hashimoto%20T%5BAuthor%5D&cauthor=true&cauthor_uid=30742731), [Shimizu T](https://www.ncbi.nlm.nih.gov/pubmed/?term=Shimizu%20T%5BAuthor%5D&cauthor=true&cauthor_uid=30742731), [Iwasa S](https://www.ncbi.nlm.nih.gov/pubmed/?term=Iwasa%20S%5BAuthor%5D&cauthor=true&cauthor_uid=30742731), [Yonemori K](https://www.ncbi.nlm.nih.gov/pubmed/?term=Yonemori%20K%5BAuthor%5D&cauthor=true&cauthor_uid=30742731), [Kato K](https://www.ncbi.nlm.nih.gov/pubmed/?term=Kato%20K%5BAuthor%5D&cauthor=true&cauthor_uid=30742731), [Morizane C](https://www.ncbi.nlm.nih.gov/pubmed/?term=Morizane%20C%5BAuthor%5D&cauthor=true&cauthor_uid=30742731), [Ogawa C](https://www.ncbi.nlm.nih.gov/pubmed/?term=Ogawa%20C%5BAuthor%5D&cauthor=true&cauthor_uid=30742731), [Tanabe N](https://www.ncbi.nlm.nih.gov/pubmed/?term=Tanabe%20N%5BAuthor%5D&cauthor=true&cauthor_uid=30742731), [Sugano K](https://www.ncbi.nlm.nih.gov/pubmed/?term=Sugano%20K%5BAuthor%5D&cauthor=true&cauthor_uid=30742731), [Hiraoka N](https://www.ncbi.nlm.nih.gov/pubmed/?term=Hiraoka%20N%5BAuthor%5D&cauthor=true&cauthor_uid=30742731), [Tamura K](https://www.ncbi.nlm.nih.gov/pubmed/?term=Tamura%20K%5BAuthor%5D&cauthor=true&cauthor_uid=30742731), [Yoshida T](https://www.ncbi.nlm.nih.gov/pubmed/?term=Yoshida%20T%5BAuthor%5D&cauthor=true&cauthor_uid=30742731), [Fujiwara Y](https://www.ncbi.nlm.nih.gov/pubmed/?term=Fujiwara%20Y%5BAuthor%5D&cauthor=true&cauthor_uid=30742731), [Ochiai A](https://www.ncbi.nlm.nih.gov/pubmed/?term=Ochiai%20A%5BAuthor%5D&cauthor=true&cauthor_uid=30742731), [Yamamoto N](https://www.ncbi.nlm.nih.gov/pubmed/?term=Yamamoto%20N%5BAuthor%5D&cauthor=true&cauthor_uid=30742731), [Kohno T](https://www.ncbi.nlm.nih.gov/pubmed/?term=Kohno%20T%5BAuthor%5D&cauthor=true&cauthor_uid=30742731). Feasibility and utility of a panel testing for 114 cancer-associated genes in a clinical setting: A hospital-based study. [Cancer Sci.](https://www.ncbi.nlm.nih.gov/pubmed/30742731) 2019 Apr;110(4):1480-1490
12. Townsley CA, Selby R, Siu LL. Systematic review of barriers to the recruitment of older patients with cancer onto clinical trials. J Clin Oncol. 2005 May 1;23(13):3112-24.
13. Yokomichi N, Morita T, Nitto A, Takahashi N, Miyamoto S, Nishie H, Matsuoka J, Sakurai H, Ishihara T, Mori M, Tarumi Y, Ogawa A. Validation of the Japanese Version of the Edmonton Symptom Assessment System-Revised. J Pain Symptom Manage. 2015;50(5):718-23.
