# Supplementary material for: Prevalence of psychological distress, quality of life, and satisfaction among patients and family members following comprehensive genomic profiling testing: Protocol of the Quality of life for Cancer genomics and Advanced Therapeutics (Q-CAT) study
Source: PLoS One. 2023 May 26;18(5):e0283968. doi: 10.1371/journal.pone.0283968 (PMC10218744; doi:10.1371/journal.pone.0283968)
Supplement: Attachment — Submitted filename: review 10.31.22.docx [file pone.0283968.s004.docx]

Review on “Prevalence of psychological distress, quality of life, and satisfaction among patients and family members following comprehensive genomic profiling testing: Protocol of the Quality of Life for Cancer genomics and Advanced Therapeutics (Q-CAT) Study”

Overall, this article has an interesting concept and the protocol sounds very interesting. Most comments are based on recommendations to better clarify sentences and improve readability.

Line 4-5: Sentence is confusing and reference needs to be moved before the period. Consider: “Comprehensive genomic profiling (CGP) and related tailored therapies guided by genomic findings, known as precision oncology, may provide cancer patients with early awaited new opportunities [2].”

Line 8: Suggest adding the word “allowing” so the sentence reads “and allowing access to clinical trials.”

Line 10-11: Sentence is confusing. Consider: “CGP testing can be used to identify different types of genomic variants, including significant variants that are actionable/druggable or variants of uncertain significance.”

Line 17-19: Sentences are confusing. Consider: “With reimbursement for CGP testing now approved, Japanese citizens have high expectations for this testing, despite the fact that actionable/druggable variants are only identified in 13.3% of samples [3].”

Line 20: Would recommend removing “Of note” and replace with “Currently,”

Line 23: place reference before the period. It should look like this: “complex area [4].”

Line 23-24: Suggest editing sentence for clarity: “While guidelines are being debated, very little is known on the knowledge cancer patients and their family members have about CGP or how they feel toward it.”

Line 27-30: Run-on sentence. Would recommend: “Only a handful of studies have explored the responses of cancer patients who have been offered CGP testing with results demonstrating patients reporting information overload and misunderstanding leading to unrealistic expectations, anxiety, and uncertainty [6-8].

Line 31-32: Would recommend replacing “non-findings” with “no identified actionable/druggable variants”.

Line 32: reference needs to come before the period. Should read “to relevant trials [6].”

Line 33: Consider removing “actually” for smoother reading

Line 34: Clarify what longitudinal data? I assume longitudinal patient reported outcome data.

Line 35-36: Suggest the following for clarity: “The availability of such data would aid the understanding of patient’s uncertainty across the genomic testing process…….”

Line 38: suggest removing “long span of four times” and replacing with “longitudinal data collection:”

Line 41: suggest removing “candidate for enough duration of time” with “the patient longitudinally,”

Line 44: can abbreviate quality of life as QOL and utilize in rest of paper.

Line 46: suggest introduction of abbreviation of electronic patient-reported outcomes (ePROs) in this line as is the first statement and then utilizing abbreviation in Line 54.

Line 56: Suggest replacing “from” to “starting”

Line 66-73: Suggest moving the aims of the study to under the heading: study design instead of setting.

Line 69: only need QOL, can delete “quality of life”

Line 90: Suggest adding “the” in front of Q-CAT staff

Line 91-93: Suggest removing the sentence starting with “Thus, at baseline they will……”

Line 93-94: Recommend only using ePRO as already defined earlier.

Line 99: Define PHQ-9 before using abbreviation

Line 99-106: run on sentence. Recommend editing to: “Additionally at each time point, selected patietns will be interviewed using the Structured Clinical Interview for DSM-V-RV (SCID), which includes items on mood disorders, neurotic, stress-related and somatoform disorders, and behavioral syndromes associated with physiological disturbances and physical factors [17-19]. Patients to be interviewed include all patients with a PHQ-9 total score of ≥ 10 and approximately 20-40 participants who score <10 as a convenience sample. These assessments will facilitate the determination of the 4 week and lifetime prevalence rates of comorbid mental disorders (Fig 1).”

Line 108: replace “be” with “include”

Line 109: no apostrophe needed after years.

Line 111: recommend “after review by the interviewer.”

Line 117: PHQ-9 was utilized earlier in Line 99 and needs to be defined there with this line only using the abbreviation.

Line 117: reference needs to go at the end of the sentence.

Line 120: need reference for the sensitivity and specificity data

Line 120: Anxiety needs to be moved as the heading of the next paragraph

Line 121: Reference needs to be at the end of the sentence

Line 123: need reference for the sensitivity and specificity data

Line 126: reference needs to go at the end of the sentence

Line 141: reference needs to go before the period.

Line 145: reference needs to go before the period.

Line 147: reference needs to go at the end of the sentence.

Line 160: reference needs to go before the period

Line 190-191: can use ePROs as the abbreviation is already identified above.

Line 209: Would recommend replacing “non-findings” with “no identified actionable/druggable variants”.

Line 211: delete extra space before the start of the sentence.

Line 212: would recommend stating “longitudinal patient report outcome data”

Line 215: Replace “electronic patient-reported outcomes” with ePROs

Line 216-217: Would recommend stating: “Study limitations include an institutional bias, as this research is performed in a single institute; however, our institute is one of the largest high volume cancer centers in Japan with higher rates of genomically matched therapy options.”

Line 221: I see you do specify longitudinal QOL data in this sentence, can utilize that specific phrase in the above comments to define the longitudinal data.

Line 223: would recommend deleting “in the real world”.

Other comments:

- Interview isn’t qualitative (which would be unstructured) but rather structured and more of a diagnostic interview. Are their plans for verifying the results and how will discrepancies be addressed?
- There is no description in the statistical section of confounding elements that affect mood, etc such as pain, other life events. How will this be accomplished?
- Need to discuss other limitations such as: 1. patients and/or family members may be lost to follow up (patient death, not coming to appointments, etc) given this is longitundinal data, thus results may be incomplete. 2. Selection bias of patients who have higher risk cancer, thus needing GCP data. These patients may have more underlying depression and anxiety than someone who has a lower risk cancer. This would speak to the generalizability of the collected data for all patients who have cancer no matter their risk. 3. The interview is based on a few interviewers, which could skew how things are documented or described. 4. Other factors affect mood, etc and with longitudinal PRO data collection, other life events can affect the results from the patient.
