# Supplementary material for: Prevalence of psychological distress, quality of life, and satisfaction among patients and family members following comprehensive genomic profiling testing: Protocol of the Quality of life for Cancer genomics and Advanced Therapeutics (Q-CAT) study
Source: PLoS One. 2023 May 26;18(5):e0283968. doi: 10.1371/journal.pone.0283968 (PMC10218744; doi:10.1371/journal.pone.0283968)
Supplement: Attachment — Submitted filename: Response to reviewers_final.docx [file pone.0283968.s005.docx]

Review on “Prevalence of psychological distress, quality of life, and satisfaction among patients and family members following comprehensive genomic profiling testing: Protocol of the Quality of Life for Cancer genomics and Advanced Therapeutics (Q-CAT) Study”

Overall, this article has an interesting concept and the protocol sounds very interesting. Most comments are based on recommendations to better clarify sentences and improve readability.

*Line 4-5: Sentence is confusing and reference needs to be moved before the period. Consider: “Comprehensive genomic profiling (CGP) and related tailored therapies guided by genomic findings, known as precision oncology, may provide cancer patients with early awaited new opportunities [2].”*

Thank you very much for your intellectual comments. We modified the sentence as advised.

<FROM>Line 4-5: Comprehensive genomic profiling (CGP) and related tailored therapies, known as precision oncology utilizing targeted agents and/or immunotherapy guided by genomic findings, may provide cancer patients with such eagerly awaited new opportunities. [2]

<TO> Line 4-5: Comprehensive genomic profiling (CGP) and related tailored therapies, known as precision oncology, may provide cancer patients with such eagerly awaited new opportunities [2].

*Line 8: Suggest adding the word “allowing” so the sentence reads “and allowing access to clinical trials.”*

Thank you very much for your important suggestions. We changed the phrase as you suggested.

<FROM>Line 8: and access to clinical trials.

<TO>Line 7: and allowing access to clinical trials.

*Line 10-11: Sentence is confusing. Consider: “CGP testing can be used to identify different types of genomic variants, including significant variants that are actionable/druggable or variants of uncertain significance.”*

We really appreciate your clear solution of confusing sentences. We used the idea.

<FROM>Line 10-11: CGP testing can be used to identify genomic variants no matter whether or not they are actionable or druggable, or of uncertain significance.

<TO> Line 9-11: CGP testing can be used to identify different types of genomic variants, including significant variants that are actionable/druggable or variants of uncertain significance.

*Line 17-19: Sentences are confusing. Consider: “With reimbursement for CGP testing now approved, Japanese citizens have high expectations for this testing, despite the fact that actionable/druggable variants are only identified in 13.3% of samples [3].”*

Thank you very much for your essential solution of confusing sentences. We changed the phrase as you suggested.

<FROM> Line 17-19: With reimbursement for CGP testing now approved, Japanese citizens have raised expectations for these tests. This anticipation is despite the fact that the actual ratio of matched therapies from the results of CGP testing is as low as 13.3% [3]

<TO> Line 16-19: With reimbursement for CGP testing now approved, Japanese citizens have high expectations for this testing, despite the fact that actionable/druggable variants are only identified in 12.2% of samples [3].

*Line 20: Would recommend removing “Of note” and replace with “Currently,”*

Thank you very much for your intellectual suggestions. We modified the sentence to your idea.

<FROM> Line 20: Of note,

<TO> Line 20: Currently,

*Line 23: place reference before the period. It should look like this: “complex area [4].”*

Thank you very much for your important comments. We would like to utilize the sentence as advised.

<FROM> Line 23: complex area. [4]

<TO> Line 23: complex area [4].

*Line 23-24: Suggest editing sentence for clarity: “While guidelines are being debated, very little is known on the knowledge cancer patients and their family members have about CGP or how they feel toward it.”*

We appreciate your clear solution of confusing sentences. We used the idea.

<FROM> Line 23-24: While debate is ongoing, very little is known about how much cancer patients and their family members know about CGP, or how they feel toward it.

<TO> Line 23-24: While guidelines are being debated, little is known about the knowledge of cancer patients and their family members have about CGP, or how they feel toward it.

*Line 27-30: Run-on sentence. Would recommend: “Only a handful of studies have explored the responses of cancer patients who have been offered CGP testing with results demonstrating patients reporting information overload and misunderstanding leading to unrealistic expectations, anxiety, and uncertainty [6-8].*

Thank you very much for your valuable suggestions. We changed the phrase as you recommended.

<FROM> Line 27-30: Only a handful of studies have explored the responses of cancer patients who have actually been offered CGP testing, and results have primarily noted patient reports of information overload and misunderstanding, which in turn lead to unrealistic expectations, anxiety and uncertainty [6–8].

<TO> Line 27-30: A very few studies have examined the responses of cancer patients recommended and underwent CGP testing with results demonstrating that patients are complaining of too much information and are misunderstanding that leads to unrealistic expectations [6–8].

*Line 31-32: Would recommend replacing “non-findings” with “no identified actionable/druggable variants”.*

Thank you very much for your intellectual suggestions. We modified the sentence as advised.

<FROM> Line 31-32: challenged by non-findings or by limited access to relevant trials. [6]

<TO> Line 31-32: challenged by no identified actionable/druggable variants, or by limitation of access to relevant genomically matched trials [6].

*Line 32: reference needs to come before the period. Should read “to relevant trials [6].”*

Thank you very much for your important suggestions. We changed to your idea.

<FROM> Line 32: trials. [6]

<TO> Line 32: trials [6].

*Line 33: Consider removing “actually” for smoother reading*

Thank you very much for your smoother suggestions. We changed the phrase as you suggested.

<FROM> Line 33: who have actually undergone CGP testing

<TO> Line 34: undergoing CGP testing

*Line 34: Clarify what longitudinal data? I assume longitudinal patient reported outcome data.*

Thank you very much for the essential question. We meant longitudinal PRO data, completely the same as your assumption, and so we used the phrase as you mentioned.

<FROM> Line 34: longitudinal data.

<TO> Line 34: longitudinal patient reported outcome data.

*Line 35-36: Suggest the following for clarity: “The availability of such data would aid the understanding of patient’s uncertainty across the genomic testing process…….”*

Thank you very much for your important suggestions. We changed to your idea.

<FROM> Line 34-36: The availability of such data would aid understanding of the experience of patient uncertainty across the genomic testing process

<TO> Line 34-35: The availability of such data would aid the understanding of patient uncertainty in the genomic testing process

*Line 38: suggest removing “long span of four times” and replacing with “longitudinal data collection:”*

Thank you very much for your intellectual suggestions. We modified the sentence as advised.

<FROM> Line 38: with long span of four times;

<TO> Line 39: with longitudinal data collection;

*Line 41: suggest removing “candidate for enough duration of time” with “the patient longitudinally,”*

Thank you very much for the essential suggestion. We changed to your modification.

<FROM> Line 40-41: By following candidate for enough duration of time,

<TO> Line 41-42: By following the patient longitudinally,

*Line 44: can abbreviate quality of life as QOL and utilize in rest of paper.*

Thank you very much for your important suggestions. We changed to your idea and utilized in rest of paper.

<FROM> Line 44: quality of life,

<TO> Line 45-46: quality of life (QOL),

*Line 46: suggest introduction of abbreviation of electronic patient-reported outcomes (ePROs) in this line as is the first statement and then utilizing abbreviation in Line 54.*

Thank you very much for your essential suggestions. We changed to your idea and utilized in rest of paper.

<FROM> Line 46: electronic patient-reported outcomes

<TO> Line 47: electronic patient-reported outcomes (ePROs),

*Line 56: Suggest replacing “from” to “starting”*

Thank you very much for the valuable suggestion. We changed to your modification.

<FROM> Line 56: Japan from May 2020.

<TO> Line 65: Japan starting May 2020.

*Line 66-73: Suggest moving the aims of the study to under the heading: study design instead of setting.*

We really appreciate your intellectual advice. We would like to move the sentences from “setting” to “study design”

<FROM> Line 66-73: The aims of this study in patients with advanced cancer undergoing CGP and their family members are as follows: …

<TO> Line 53-60: The aims of this study in patients with advanced cancer undergoing CGP and their family members are as follows:…

*Line 69: only need QOL, can delete “quality of life”*

Thank you very much for the important suggestion. We changed to your idea.

<FROM> Line 69: quality of life (QOL),

<TO> Line 56: QOL,

*Line 90: Suggest adding “the” in front of Q-CAT staff*

Thank you very much for the valuable suggestion. We changed to the phrase according to as you suggested.

<FROM> Line 90: for the study to Q-CAT staff

<TO> Line 92: for the study to the Q-CAT staff

*Line 91-93: Suggest removing the sentence starting with “Thus, at baseline they will……”*

We really appreciate your essential advice. We would like to remove the sentences.

<FROM> Line 91-93: Thus, at baseline they will have already considered and given consent for CGP testing but will not have received the results of CGP testing.

<TO> Removed

*Line 93-94: Recommend only using ePRO as already defined earlier.*

Thank you very much for the important insight. We changed to your recommendation.

<FROM> Line 93-94: an electronic patient reported outcome (ePRO)

<TO> Line 93: an ePRO

*Line 99: Define PHQ-9 before using abbreviation*

Thank you very much for the valuable suggestion. We added to the phrase according to as you

<FROM> Line 99: a PHQ-9 score

<TO> Line 104-105: a Patient Health Questionnaire (PHQ-9) total score

*Line 99-106: run on sentence. Recommend editing to: “Additionally at each time point, selected patietns will be interviewed using the Structured Clinical Interview for DSM-V-RV (SCID), which includes items on mood disorders, neurotic, stress-related and somatoform disorders, and behavioral syndromes associated with physiological disturbances and physical factors [17-19]. Patients to be interviewed include all patients with a PHQ-9 total score of ≥ 10 and approximately 20-40 participants who score <10 as a convenience sample. These assessments will facilitate the determination of the 4 week and lifetime prevalence rates of comorbid mental disorders (Fig 1).”*

We really appreciate your essential advice. We would like to modify our sentence to your idea.

<FROM>Line 99-106: All patients with a PHQ-9 score at the cutoff point of 10 or above (total score) [17,18] as well as a small subset of participants (around 20–40) selected as a convenience sample who score below this cutoff point will be interviewed used the Structured Clinical Interview for DSM-V-RV (SCID), which includes items on mood disorders, neurotic, stress-related and somatoform disorders, and behavioral syndromes associated with physiological disturbances and physical factors [19 ], at each assessment time-point to determine the 4-week and lifetime prevalence rates of comorbid mental disorders (Fig. 1).

<TO>Line 100-107: Additionally at each time point, selected patients will be interviewed used the Structured Clinical Interview for DSM-V-RV (SCID), which is a semi-structured interview for making the major *DSM-5* diagnoses such as mood disorders, neurotic, stress-related and somatoform disorders, and behavioral syndromes associated with physiological disturbances and physical factors [17-19]. Patients to be interviewed include all patients with a Patient Health Questionnaire (PHQ-9) total score of > 10 and approximately 20-40 participants who score <10 as a convenience sample. The 4 week and lifetime prevalence rates of comorbid psychiatric disorders will be determined easier by this scale (Fig 1).

*Line 108: replace “be” with “include”*

*Line 109: no apostrophe needed after years.*

*Line 111: recommend “after review by the interviewer.”*

Thank you very much for the important comments. We replaced according to your recommendations.

<FROM> Line 108: will be two clinical psychologists, Line 109: 21 years’, Line 111: confirmed by review by the interviewers.

<TO> Line 109: will include two clinical psychologists, Line 110: 21 years, Line 116: confirmed after review by the interviewers.

*Line 117: PHQ-9 was utilized earlier in Line 99 and needs to be defined there with this line only using the abbreviation.*

*Line 117: reference needs to go at the end of the sentence.*

*Line 120: need reference for the sensitivity and specificity data*

We truly appreciate your valuable suggestions. We used the abbreviation and modified the position of reference at the bottom of the paragraph as the sensitivity and specificity data is from reference [9].

<FROM> Line 117-120: The nine items of Patient Health Questionnaire-9 (PHQ-9) [9] will be used to measure depression. Higher scores indicate greater possibility of major depression (a PHQ-9 score ≥10, the cut off score, had a sensitivity of 88% and a specificity of 88% for major depression).

<TO> Line 122-124: The nine items of PHQ-9 will be used to measure depression. Higher PHQ-9 scores indicate greater possibility of major depression (a PHQ-9 score ≥10, the cut off score, had a sensitivity of 88% and a specificity of 88% for major depression) [9].

*Line 120: Anxiety needs to be moved as the heading of the next paragraph*

*Line 121: Reference needs to be at the end of the sentence*

*Line 123: need reference for the sensitivity and specificity data*

We really appreciate your essential suggestions. We moved the Anxiety and modified the position of reference at the bottom of the paragraph as the sensitivity and specificity data is from reference [10].

<FROM> Line 120-123: Anxiety

The seven items of General Anxiety Disorder-7 (GAD-7) [10] will be used to measure anxiety. Higher scores indicate greater possibility of generalized anxiety disorder (a GAD-7 score ≥10 had a sensitivity of 89% and specificity of 82% for generalized anxiety disorder).

<TO> Line 125-128: Anxiety

The seven items of General Anxiety Disorder-7 (GAD-7) will be used to measure anxiety. Higher scores indicate greater possibility of generalized anxiety disorder (a GAD-7 score ≥10 had a sensitivity of 89% and specificity of 82% for generalized anxiety disorder) [10].

*Line 126: reference needs to go at the end of the sentence*

*Line 141: reference needs to go before the period.*

*Line 145: reference needs to go before the period.*

*Line 147: reference needs to go at the end of the sentence.*

*Line 160: reference needs to go before the period*

Thank you very much for the important comments. We modified the positions of references.

*Line 190-191: can use ePROs as the abbreviation is already identified above.*

*Line 209: Would recommend replacing “non-findings” with “no identified actionable/druggable variants”.*

We really appreciate your essential advice. We would like to modify our sentence to your idea.

<FROM> Line 190-191: using electronic patient-reported outcomes (ePROs)., by non-findings or by limited access to relevant trials.

<TO> Line 198: using ePROs., Line 218: by no identified actionable/druggable variants or by limited access to genomically matched trials.

*Line 211: delete extra space before the start of the sentence.*

*Line 212: would recommend stating “longitudinal patient report outcome data”*

*Line 215: Replace “electronic patient-reported outcomes” with ePROs*

Thank you very much for the important comments. We replaced according to your recommendations.

<FROM> Line 211-215: To our knowledge, none of the studies to date of patients who have actually undergone CGP testing have reported longitudinal data which would allow an understanding of the experience of patient uncertainty across the CGP testing process and the long-term psychosocial impact on the patients. The Q-CAT study constitutes a psycho-social/ethical study to gather electronic patient-reported outcomes and qualitative data.

<TO> Line 220-224: To our knowledge, no previous studies of patients underwent CGP testing have reported longitudinal patient reported outcome data which would allow an understanding of the experience of patient uncertainty across the CGP testing process and the long-term psychosocial impact on the patients. The Q-CAT study constitutes a psycho-social/ethical study to gather ePROs and qualitative data.

*Line 216-217: Would recommend stating: “Study limitations include an institutional bias, as this research is performed in a single institute; however, our institute is one of the largest high volume cancer centers in Japan with higher rates of genomically matched therapy options.”*

We really appreciate your valuable recommendation. We would like to modify our sentence to your idea.

<FROM> Line 216-217: We also admit study limitation on institutional bias, as this research is performed in a single institute, which is one of the largest high volume cancer centers in Japan.

<TO> Line 216-217:

*Line 221: I see you do specify longitudinal QOL data in this sentence, can utilize that specific phrase in the above comments to define the longitudinal data.*

*Line 223: would recommend deleting “in the real world”.*

Thank you very much for the important insight. We defined into the longitudinal ePROs data and deleted the phrase along with your recommendation.

<FROM> Line 221-223: This will be the first Japanese study to collect longitudinal QOL data via ePRO on the experience of cancer patients and their family members with comprehensive genomic profiling in the real world.

<TO> Line 238-239: This will be the first study in Japan to longitudinally collect QOL data via ePRO on the experience of cancer patients and their family members with CGP.

Other comments:

- *Interview isn’t qualitative (which would be unstructured) but rather structured and more of a diagnostic interview. Are their plans for verifying the results and how will discrepancies be addressed?*
- *There is no description in the statistical section of confounding elements that affect mood, etc such as pain, other life events. How will this be accomplished?*
- We would like to show our best gratitude for giving us important idea. We would like to put additional paragraph in “Interviewer training and quality standards” session to discuss the verification and approach toward possible discrepancies. Additionally, we put a paragraph in “Statistical method and Data analysis” session to discuss the verification and approach toward possible discrepancies.

<FROM> No description, Line 175-181: Temporal changes in scales will be investigated by calculating differences between time-points. Comparisons of demographic and other factors will be conducted using the t-test and ANOVA, and Kruskal-Wallis test. Associations between variables will be examined using correlation analysis, chi-squared tests and regression analysis, depending on the presence or absence of depression and anxiety based on outcome scores. Multiple or logistic regression analysis will be used to adjust for the effects of confounders and to identify predictors of outcome.

<TO> Line 112-115: The interviews will be performed in each combination with two of the three interviewers present, who both independently scored the answers until the kappa values will be 1.0 for all diagnosis of the interviews, indicating substantial agreement of all diagnoses between the two interviewers., Line 182-189: Bivariate associations and comparisons of the outcome scores, depression, anxiety and QOL, with variables such as knowledge, satisfaction with oncologist, quality of communication, symptoms, demographic factors, disease details, social support, and the results of CGP testing, will be conducted using correlation analysis, chi-squared tests, t-tests and ANOVA, and Kruskal-Wallis tests. In order to adjust for the effects of confounding factors, and to identify predictive factors of outcome, multivariate associations between variables will be conducted using Multiple or logistic regression analysis with depending on the outcomes.

- *Need to discuss other limitations such as:*
- *1. patients and/or family members may be lost to follow up (patient death, not coming to appointments, etc) given this is longitudinal data, thus results may be incomplete.*
- *2. Selection bias of patients who have higher risk cancer, thus needing GCP data. These patients may have more underlying depression and anxiety than someone who has a lower risk cancer. This would speak to the generalizability of the collected data for all patients who have cancer no matter their risk.*
- *3. The interview is based on a few interviewers, which could skew how things are documented or described.*
- *4. Other factors affect mood, etc and with longitudinal PRO data collection, other life events can affect the results from the patient.*

Thank you very much for your valuable, essential, and critical comments. We would like to put additional paragraph in Discussion session to discuss the limitations to answer the comments.

<FROM> Line 216-220: We also admit study limitation on institutional bias, as this research is performed in a single institute, which is one of the largest high volume cancer centers in Japan. Therefore, compared with multicenter study, outcome of single center study will be more vulnerable to the risk of bias. Furthermore, access rate of genomically matched therapy would be highest in the country.

<TO> Line 225-237: Study limitation includes an institutional bias, as this research is performed in a single institute; however, our institute is one of the largest high volume cancer centers in Japan with higher rates of genomically matched therapy options. Moreover, patients and/or family members may be lost to follow up (patient death, not coming to appointments, etc) given this research is collecting longitudinal ePROs data, thus results may be incomplete. Even though we didn’t limit cancer types, there might be a possibility of having selection bias of patients who have higher risk cancer, thus needing GCP data. These patients may have more underlying depression and anxiety than someone who has a lower risk cancer. This would speak to the generalizability of the collected data for all patients who have cancer no matter their risk. In order to approach this issue, analysis by cancer types might be helpful to understand the backgrounds. Some elements may become confounding factors that affect the mood during the interviews, such as symptoms and other life events during the clinical course.
